# Functional characterization and phenotyping of RAB2A and Lactadherin/MFGE8 as boar sperm zona pellucida binding proteins

**DOI:** 10.3389/fcell.2025.1653053

**Published:** 2025-10-02

**Authors:** Natalie Zelenkova, Veronika Kraus, Lukas Ded, Daniela Spevakova, Lucie Sadilkova, Michaela Frolikova, Aneta Pilsova, Zuzana Pilsova, Barbora Klusackova, Ondrej Simonik, Eva Chmelikova, Tereza Krejcova, Michal Zigo, Peter Sutovsky, Marketa Sedmikova, Katerina Komrskova, Pavla Postlerova

**Affiliations:** ^1^ Department of Veterinary Sciences, Faculty of Agrobiology, Food and Natural Resources, Czech University of Life Sciences, Prague, Czechia; ^2^ Laboratory of Reproductive Biology, Institute of Biotechnology of the Czech Academy of Sciences, BIOCEV, Vestec, Czechia; ^3^ Division of Animal Sciences, College of Agriculture, Food and Natural Resources, University of Missouri, Columbia, MO, United States; ^4^ Department of Obstetrics, Gynaecology and Women’s Health, University of Missouri, Columbia, MO, United States; ^5^ Department of Zoology, Faculty of Science, Charles University, Prague, Czechia

**Keywords:** sperm-ZP interaction, antibody-blocking, competitive binding assays, p47, SED1, acrosome, capacitation, sperm-oocyte

## Abstract

Mammalian fertilization begins with the species-specific binding of spermatozoa to the oocyte’s zona pellucida (ZP), a process mediated by multiple surface proteins forming a functional receptor complex. Among them, the Ras oncogene family protein RAB2A and lactadherin/MFGE8 (p47/SED1) were previously identified as ZP-binding candidates in pig; however, their functional roles in sperm-oocyte interactions remained unconfirmed. This study aimed to evaluate their involvement in sperm-ZP binding by using antibody-blocking and competitive binding assays with porcine oocytes. In parallel, we validated the specificity and functional relevance of two in-house raised monoclonal antibodies, 5C5 and 1H9, targeting RAB2A and lactadherin/MFGE8, respectively, and further characterized these proteins in boar spermatozoa. Immunofluorescence detection indicated that both RAB2A and lactadherin/MFGE8 became accessible on the sperm surface upon capacitation. Their surface localization at this stage supports their potential involvement in the primary sperm-ZP interactions preceding acrosomal exocytosis. Moreover, the sperm-specific 5C5 antibody detected reduced RAB2A levels in ejaculates from men with abnormal sperm parameters. This highlights the potential of RAB2A as a biomarker of sperm quality and acrosomal integrity, with promising translational relevance from animal models to humans. *In vitro* sperm-zona binding assays revealed that while in-house raised antibody treatments showed no significant inhibition, follow-up experiments using a commercial anti-RAB2A antibody demonstrated a significant reduction in sperm binding to the ZP of the oocyte. Both recombinant RAB2A (rc-RAB2A) and recombinant lactadherin (rc-lactadherin) significantly reduced sperm-ZP binding, highlighting their functional relevance. Our results support the role of RAB2A and lactadherin/MFGE8 in sperm-oocyte binding and highlight the utility of monoclonal antibodies 5C5 and 1H9 for sperm phenotyping. Future work should focus on identifying molecular interaction partners and signaling mechanisms that mediate the initiation of acrosomal exocytosis at fertilization.

## 1 Introduction

Mammalian fertilization is a highly orchestrated biological process that begins with the recognition and binding of spermatozoa to the zona pellucida (ZP), an extracellular glycoprotein matrix surrounding the oocyte. This initial interaction, mediated by specific proteins on the surfaces of both gametes, is critical for the species-specific gamete binding and essential for successful fertilization, as it enables the spermatozoon to recognize, adhere to, and penetrate the ZP, ultimately leading to fusion with the oocyte plasma membrane, the oolemma (Yanagimachi, 1994). Sperm ZP receptors involved in the primary binding are localized to the apical region of the sperm head plasma membrane (van Gestel et al., 2007; Kongmanas et al., 2015).

The earlier assumption that a single receptor molecule is responsible for the interaction of the spermatozoa with the ZP of the oocyte has been disproven. On the contrary, there is now a preponderance of evidence suggesting the coordinated action of multiple receptor molecules forming the functional multimeric zona-binding complexes (Tanphaichitr et al., 2007; Redgrove et al., 2011). Such a complex network of zona receptors could increase the likelihood of successful sperm binding to the ZP, thereby enhancing the chances of fertilization. This model may also explain the presence of numerous sperm receptor proteins that have been identified as playing a role in sperm interaction with the ZP (van Gestel et al., 2007; Redgrove et al., 2012). A significant number of sperm head plasma membrane proteins with an affinity for ZP have been described (reviewed in Tumova et al., 2021), and the list of these receptors is periodically revisited as new potential proteins for primary binding are identified, while the significance of others is reevaluated (van Gestel et al., 2007; Tanphaichitr et al., 2015). These sperm receptors cluster together to form high-molecular-weight complexes that appear to contain a variety of proteins with different functions, including chaperones, enzymes, such as kinases and proteases, signaling proteins, and cell adhesion proteins that mediate the sperm interaction with the ZP (Kongmanas et al., 2015). The zona-binding complexes have been found assembling as components of so-called lipid rafts in the anterior head plasma membrane. These ZP-binding complexes undergo remodeling and redistribution during sperm capacitation and are predominantly localized to the apical region of the capacitated sperm head (Tanphaichitr et al., 2007; Nixon and Aitken, 2009; Kongmanas et al., 2015).

Precise characterization of sperm proteins involved in the species-specific sperm-ZP interaction is crucial for improving diagnostic and reproductive technologies in animals as well as humans. To study the localization and functional relevance of two proposed boar sperm-ZP receptors, we employed two in-house generated monoclonal antibodies that were prepared by immunizing mice with isolated boar sperm surface proteins. The proteins targeted by these antibodies were previously identified as the Ras-family protein RAB2A and lactadherin/MFGE8 by using immunoprecipitation and MALDI-TOF mass spectrometry of boar sperm extract (Zigo et al., 2015). While the role of RAB2A in sperm physiology is not fully understood, it is believed to participate in the regulation of capacitation, acrosome reaction, and sperm motility, and is proposed to have utility as a male fertility biomarker (Kwon et al., 2015; Bae et al., 2019; Bae et al., 2022; Zigo et al., 2024). Additionally, it has been linked to acrosome formation in bull spermatozoa (Mountjoy et al., 2008), where it was localized to the cytosolic face of the forming acrosome in the spermatogenic cells. However, Zigo et al. (2015) later reported the presence of RAB2A on the surface of boar spermatozoa and proposed its possible involvement in the sperm-ZP binding. Lactadherin/MFGE8 is a peripheral membrane protein also known as p47, which is homologous to mouse SED1. The role of this protein in sperm-ZP recognition and binding has been known in mice (Ensslin and Shur, 2003). In porcine spermatozoa, lactadherin/MFGE8 has been shown to mediate the adhesion to the oviduct epithelium, participating in the formation of the sperm reservoir (Silva et al., 2017). Both of these proteins have been proposed as candidate sperm-ZP receptors in pigs (Ensslin et al., 1998; Van Gestel et al., 2007; Zigo et al., 2015), but to date, no functional studies employing binding assays using intact porcine gametes to validate this prediction have been published. Tanphaichitr et al. (2015) also recognized lactadherin/MFGE8 as one of the boar sperm proteins that interact to form high-molecular-weight complexes in the apical plasma membrane with binding affinity to the ZP. Lack of verification that these proteins are indeed involved in direct sperm-oocyte interactions is a recognized knowledge gap in fertilization biology.

Both RAB2A and lactadherin/MFGE8 proteins exhibited affinity towards solubilized ZP glycoproteins in a previous study (Zigo et al., 2015). The present study was designed to functionally evaluate the involvement of RAB2A and lactadherin/MFGE8 in sperm-ZP binding by using antibody-blocking and competitive binding assays performed directly on porcine oocytes. In parallel, we focused on validating the specificity and functional relevance of the in-house raised monoclonal antibodies 5C5 and 1H9 on spermatozoa directly, as well as on further characterization of the corresponding proteins in mammalian sperm physiology.

## 2 Materials and methods

### 2.1 Antibodies and recombinant proteins

The primary anti-RAB2A (5C5) and anti-lactadherin (1H9) antibodies used in this study are mouse monoclonal antibodies produced in-house by immunizing mice with an isolated boar sperm surface protein fraction. The proteins recognized by these antibodies were identified using MALDI-TOF (Zigo et al., 2015). The anti-lactadherin/1H9 antibody was characterized in the study by Zigo et al. (2019), and the specificity of the 5C5 antibody against RAB2A was previously confirmed by Zigo et al. (2024) using a blocking peptide assay with recombinant human RAB2A. Similarly, the specificity of the 1H9 antibody was verified in this present study by Western blot analysis, demonstrating its ability to detect recombinant pig lactadherin/MFGE8 (Supplementary Figure S1). IgG concentrations in hybridoma supernatants (5C5, 1H9) were quantified by ELISA using a mouse IgG standard (Abcam, UK). Serially diluted supernatants were coated onto a microtiter plate. After incubation with anti-mouse IgG conjugated with horse radish peroxidase (#1706516, Goat Anti-Mouse IgG (H + L)-HRP Conjugate, Bio-Rad, USA) and TMB (3,3′,5,5′-Tetramethylbenzidine) substrate (#T0440), absorbance at 449 nm was measured in Microplate reader (Tecan, Austria). Concentrations were calculated via standard curve using GainData software (Arigo, Taiwan). Based on ELISA measurements, antibody concentrations were determined as 0.35 μg/mL for 5C5 and 0.45 μg/mL for 1H9 antibodies. The isotype control (mouse IgG Isotype Control, ThermoFisher Scientific, USA) was used in our experiment at a higher concentration (1 μg/mL) to ensure specificity and reveal any non-specific IgG binding. Alternatively, a commercial rabbit polyclonal antibody anti-RAB2A (#PA5-101823, ThermoFisher Scientific) was used for the binding assays and was validated based on the immunofluorescent localization of RAB2A in boar spermatozoa (Supplementary Figures S2, S3). For immunofluorescence microscopy, secondary anti-mouse (#A32723, Goat anti-Mouse IgG (H + L) Highly Cross-Adsorbed Secondary Antibody, Alexa Fluor™ Plus 488, Invitrogen, USA) and anti-rabbit (Goat anti-Rabbit IgG (H + L) Cross-Adsorbed Secondary Antibody, Alexa Fluor™ 488, #A11001, Invitrogen, USA) antibodies were used. For Western blot analysis, secondary anti-mouse antibody (#1706516, Goat Anti-Mouse IgG (H + L)-HRP Conjugate) was employed. For antibody specificity verification and competitive binding assays, recombinant human RAB2A (#PR0373; 6×His-GST tagged; Canvax Biotech, S.L., Spain) and pig lactadherin/MFGE8 (#CSB-EP013752PI; C-terminal 6×His-tagged; Cusabio Technology LLC, USA) were utilized. Unless otherwise stated, all chemicals used in this study were purchased from Sigma-Aldrich (USA).

### 2.2 Semen, ovaries, and tissue collections

Semen collected from fertile Duroc boars bred (12–18 months old) for commercial artificial insemination was provided by the Insemination Station Skrsin (LIPRA PORK a.s., Rovensko pod Troskami, CR). The handling and care of animals were in accordance with Council Directive 98/58/EC, Act No. 154/2000 Coll., and Act No. 246/1992 Coll. of the Czech National Council. Fresh ejaculates were processed immediately upon receipt and used for immunofluorescent localization and Western blot analysis. Extended insemination doses were stored up to 2 days in a cooling box at 17 °C for subsequent use in sperm-ZP binding assays. The male reproductive tissues (testes and epididymides) were obtained from sexually mature Prestice Black-Pied boars, 8–12 months old, from the Biofarm Sasov (Jihlava, CR) under ethical conditions, in compliance with the relevant animal welfare laws and regulations, specifically Directive 2010/63/EU and Czech legislation (Directive 208/2004 Sb.). After collecting, the organs were transported on ice and processed immediately. Ovaries from slaughtered young gilts (Landrace or Czech Large White) were obtained from a slaughterhouse in Pribram (CR), and transported to the laboratory in a thermobox at 38.5 °C. Cumulus-oocyte complexes isolated from ovaries were processed immediately.

Human spermatozoa were obtained from the ejaculate of men after 3–4 days of sexual abstinence, and processed in the Immunological Laboratory, Gennet (Prague, CR). Sample collection from consenting, de-identified donors, was approved by the Ethics Committee of the General University Hospital, Prague (671/17 S-IV) with informed consent and in accordance with the institute’s Human Ethics Committee guidelines. Ejaculates with normospermic parameters (NS), ejaculates with reduced sperm motility–asthenozoospermia (AS), ejaculates with reduced sperm concentration–oligozoospermia (OS), and ejaculates with reduced sperm concentration and motility and increased morphological abnormalities–oligoasthenoteratozoospermia (OAT) were used in the study, with classification based on the WHO manual (World Health Organization, 2021), according to which normozoospermia is defined as ejaculate volume ≥1.4 mL, sperm concentration ≥16 million/mL, total sperm count ≥39 million, total motility ≥42%, viability ≥54%, and normal morphology ≥4%. A total of four semen samples were analyzed for each spermiogram category.

### 2.3 Oocyte *in vitro* maturation

Cumulus-oocyte complexes (COCs) were aspirated from ovarian follicles of 2–6 mm diameter by using a needle and syringe and washed three times in phosphate-buffered saline (PBS; #P4417) containing 0.2 g/L polyvinyl alcohol (PBS-PVA). Only good-quality COCs with compact oocyte cytoplasm and fully surrounded by cumulus cells were selected during the washing process and then processed as described in Navarro-Serna et al. (2021). Briefly, selected COCs were washed in NCSU-37 maturation medium (108.73 mM NaCl, 25.07 mM NaHCO_3_, 4.78 mM KCl, 1.19 mM KH_2_PO_4_, 1.19 mM MgSO_4_, 1.7 mM CaCl_2_, 5.55 mM D-glucose, 12 mM D-sorbitol, 0.18 mM penicillin, 39 mM streptomycin sulphate). Groups of 50–55 COCs were cultured in 500 µL of NCSU-37 supplemented with 0.57 mM cysteine, 50 µM β-mercaptoethanol, 5.00 mg/L insulin, 1 mM dibutyryl cAMP, 10 IU/mL equine chorionic gonadotropin (eCG), 10 IU/mL human chorionic gonadotropin (hCG), and 10% (v/v) porcine follicular fluid at 38.5 °C and 5% CO_2_ for 22 h. Then, oocytes were washed in fresh NCSU-37 medium without dibutyryl cAMP, eCG, and hCG and cultured for an additional 20–22 h.

### 2.4 Epididymal sperm isolation

To isolate the sperm cells, individual parts of the epididymis (caput, corpus, and cauda) from sacrificed boars from slaughterhouse were excised and small tissue sections were incubated in PBS solution at 38 °C for 30 min, allowing spermatozoa to be released into the solution, which was then filtered through a 15 µm mesh and centrifuged for 5 min at 50×g, and the supernatant containing spermatozoa were collected. The spermatozoa were washed 3× in PBS and centrifuged at 500 *g* for 10 min. Finally, the sperm concentration was adjusted to 5 × 10^7^ sperm/mL, and the samples were used for the experiments described below.

### 2.5 Tissue section preparation

Small pieces of boar testes and individual parts of epididymides were embedded in TissueTek (OCT Compound for Cryostat Sectioning; Sakura Finetek, Netherlands) and carefully immersed in liquid nitrogen. Then they were processed into cryosections on a Leica Cryocut (Leica, Germany). Cryosections with a thickness of 5 µm were captured on slides and were further stored at −20 °C. For fixation, 100 μL of ice-cold acetone was applied to each immobilized section, and the slides were left to fix for 30 min. Then, they were rinsed with PBS and dried. The prepared cryosections were then used for indirect immunofluorescence microscopy.

### 2.6 Semen processing

Fresh boar ejaculates were centrifuged at 300 *g* for 10 min to remove the seminal plasma and/or extenders. Spermatozoa were washed three times with PBS and centrifuged again at 300×g for 10 min. The sperm pellet was resuspended, and the sperm concentration was assessed and adjusted to 5 × 10^7^ sperm/mL. Spermatozoa without seminal plasma were *in vitro* capacitated in Tyrode’s albumin lactate pyruvate medium (TALP; Matás et al., 2010) in the incubator at 38.5 °C and 5% CO_2_ for 3 h. Next, samples were washed in PBS-PVA. After washing, the sperm concentration was checked again and adjusted to 5 × 10^7^ sperm/mL. For the *in vitro* induction of acrosomal exocytosis, the calcium ionophore A 23187 (#C7522) was added to the sperm sample at a final concentration of 10 μM, and the spermatozoa were incubated at 38 °C and 5% CO_2_ for 1 h. The samples at each stage of sperm maturation were used for immunofluorescence and Western blotting.

Cryopreserved human ejaculates were thawed by incubation in a water bath at 37 °C for 10 min. After liquefaction, the spermatozoa were separated from seminal plasma by gradient centrifugation (55%/80%; SupraSperm®System, Origio, Denmark) at 300×g for 25 min at 37 °C. The selected spermatozoa were then washed twice in Sperm Wash medium (Origio), centrifuged at 300 *g*for 10 min, and finally resuspended in 1 mL of saline prewarmed to 37 °C. To the washed pellet of ejaculated sperm, 1 mL of Sperm Preparation medium (Origio) preheated to 37 °C was added, and the samples were *in vitro*capacitated in this medium for 2 h in a 5% CO_2_and at 37 °C. After incubation, the samples were centrifuged at 300 *g*for 10 min and then washed in Sperm Wash medium and further processed. After *in vitro*capacitation, calcium ionophore at a final concentration of 10 μM was added, and spermatozoa were incubated at 37 °C and 5% CO_2_for 1 h. The samples were centrifuged again at 300×g for 10 min and washed in Sperm Wash medium. Finally, the sperm concentration was adjusted to 5 × 10^7^sperm/mL, and samples were used for experiments.

### 2.7 Immunofluorescence microscopy

For immunofluorescence microscopy, 20 µL of the sperm suspension, prepared as described above and adjusted to a concentration of 1 × 10^7^ sperm/mL was pipetted onto the microscopy slide. The sperm suspension was covered with 50 µL of an ice-cold acetone-methanol mixture (1:1) to fix and permeabilize the sample. After 5 min of incubation, the samples were rinsed with PBS and left to dry. Prepared slides were either stored in a refrigerator at 4 °C or used immediately for the immunofluorescence experiment. First, 150 µL of SuperBlock blocking medium was applied to each sample. The slides were then transferred to a dark, humid chamber and incubated for 30 min at room temperature (RT). After incubation, the slides were rinsed with PBS and dried. Subsequently, 100 µL of mouse monoclonal antibodies (anti-RAB2A/5C5, anti-lactadherin/1H9) diluted at 1:2, or commercial anti-RAB2A rabbit polyclonal antibody diluted in PBS (20 μg/mL) was added to each sample. The negative control samples were covered with 100 µL PBS. All samples were then incubated overnight at 4 °C in a humid chamber. The next day, the samples were washed with PBS and incubated with 100 µL of a secondary anti-mouse or anti-rabbit antibody with fluorochrome-conjugated immunoglobulin diluted in PBS at a 1:300 ratio. After incubation for 1 h at 4 °C, the samples were washed with PBS and dried. To check the presence/integrity of the acrosomes, 100 µL lectin PNA conjugated with rhodamine (Rhodamine Peanut Agglutinin; Vector Laboratories, Inc., USA) diluted at 1:1,000 in PBS was added to the samples and incubated in a humid chamber for 30 min at RT. After incubation, the samples were washed with PBS and subsequently with distilled water. The samples were dried, and 5 µL of mounting medium with DAPI was applied to the slides (VectaShield Antifade Mounting Medium with DAPI; Vector Laboratories, Inc.) to counterstain the cell nuclei. Samples were then covered with coverslips and evaluated under an epifluorescence microscope (ECLIPSE Ni, Nikon, Japan) with ×600 magnification, and images were captured using NIS software (Nikon). For higher resolution, samples were evaluated under a confocal microscope (Zeiss, Germany) at ×600 magnification, and images were captured using ZEN software (Zeiss). Alternatively, to test the protein localization in native cells, proteins were immunolocalized in live spermatozoa prior to fixation and permeabilization. Primary antibodies were applied directly to a washed sperm suspension and incubated for 2 h at 38 °C on a rocking platform. Following this, the sperm suspensions were washed twice with PBS, resuspended, and pipetted onto the slides and covered with 50 µL of an ice-cold acetone-methanol mixture (1:1) for 5 min. The slides were then washed and blocked for non-specific binding with 150 µL of SuperBlock blocking medium for 30 min. The remaining steps, including incubation with secondary antibodies, acrosome staining with PNA lectin, and mounting with DAPI-containing medium for nuclear labeling were carried out as described above. For the preparation of samples for SIM (Structured Illumination Microscopy), the same procedure as for the confocal microscopy was used, with a modification such that the spermatozoa were fixed on the coverslips (thickness No. 1.5 H, 170 ± 5 μM, Paul Marienfeld GmbH and Co. KG, Germany) and Hoechst 33342 at a concentration of 1:100 was used for visualization of nuclei. Finally, air-dried samples were fixed with 90% (v/v) glycerol with 5% (v/v) N-propyl gallate.

### 2.8 Boar sperm membrane fractionation

Membrane fractionation was performed according to Somanath and Gandhi (2004), modified for bull and boar spermatozoa (Antalíková et al., 2015; Palenikova et al., 2021). Fresh boar ejaculate (15 mL) was diluted 1:2 with Krebs Ringer Bicarbonate (KRB) medium: 0.7 mM Na_2_HPO_4_, 0.49 mM MgCl_2_, 4.56 mM KCl, 0.1198 M NaCl, 0.0013 M NaH_2_PO_4_, 2.37 mM fructose, 0.0149 M NaHCO_3_ (pH 7.4). Diluted boar semen was layered on top of the solution of 1.3 M sucrose with 0.9% (w/v) NaCl and centrifuged for 30 min at 2,000×g at 4 °C. Sperm pellet was resuspended in 0.15 M NaCl with 5 mM HEPES (pH 7.0), layered on top of1.3 M sucrose with 0.9% (w/v) NaCl and ultracentrifuged for 20 min at 34,000×g (Beckman, Canada) at 4 °C. The pellets were resuspended in 0.15 M NaCl with 5 mM HEPES (pH 7.0) supplemented with protease inhibitors Complete Mini (ThermoFisher Scientific) and sonicated in 10 × 10 s intervals (Ultrasonic, 80 Amplitude microns power). Membranes from homogenate were separated by a discontinuous sucrose gradient consisting of 1.75 M sucrose and 1.3 M sucrose (1:1) and ultracentrifugation for 3 h at 95,000×g at 4 °C. The plasma membrane (PM) fraction was recovered from the interface between the sample and 1.3 M sucrose. The outer acrosomal membrane (OAM) fraction, together with tails, was at the interface between 1.3 M/1.75 M sucrose. The pellet contained inner acrosomal membranes (IAM) and the remaining equatorial segments, which were closely associated with the sperm heads (Somanath and Gandhi, 2004). The fractions of PM and OAM were diluted with PBS and pelleted by ultracentrifugation for 30 min at 120,000×g at 4 °C. The fraction of IAM was washed with PBS and centrifuged at 3,500×g for 10 min at 4 °C. Subsequently, the separated membrane fractions were solubilized with 1% (v/v) Triton X-100 for 1 h at 4 °C, and proteins were precipitated with 6-fold ice-cold acetone. Precipitated proteins were then incubated at 95 °C for 5 min in 2× concentrated reducing Laemmli sample buffer (20% (v/v) glycerol, 4% (w/v) sodium dodecyl sulfate (SDS), 0.005% (w/v) bromophenol blue, 0.125 M Tris-HCl, pH 6.8), supplemented with β-mercaptoethanol to a final concentration of 5% (v/v).

### 2.9 Isolation of perinuclear theca (PT)

The isolation of PT was performed according to Mountjoy et al. (2008). Fresh boar or human ejaculates were centrifuged at 500 *g*and spermatozoa were washed twice in 25 mM Tris-buffered saline (TBS) (pH 7.6) at 4 °C. Spermatozoa were diluted in TBS buffer with protease inhibitors 10:1 (v/v) and the sperm suspension was sonicated on ice for 10 × 10 s intervals (Ultrasonic, 80 Amplitude microns power), until 99% of the sperm had separated the heads from the tails. The sonicated spermatozoa were washed twice in TBS at 500×g at 4 °C. The final sperm pellet was diluted in TBS, and the sperm suspension was laid over the 80% (w/v) sucrose. Samples were ultracentrifuged in a SW40Ti centrifuge with a swinging-bucket rotor at 50,000 rpm, 65 min (Beckman). After centrifugation, sperm heads were stranded on the outside-facing wall of the tube, and tails were on the inside-facing wall of the tube. The head pellet was washed twice in TBS and incubated with 0.2% (v/v) Triton X-100 for 1 h on ice with periodic vortexing. The pellet was incubated with 1 M KCl on ice for 1 h with periodic vortexing, followed by centrifugation (2,500×g, 10 min) to obtain the supernatant containing the ion-bound PT proteins (PTI). Next, 100 mM NaOH was added to the rest of the pellet, and samples were incubated overnight with constant spinning, and the supernatant containing covalently bound PT proteins (PTC) was collected after centrifugation. The final pellet was extracted with 2× Laemmli sample buffer on ice for 30 min and centrifuged (10,000×g, 2 min) to collect remaining head proteins. All supernatants were precipitated with 6 volumes of ice-cold acetone, then dissolved in 2× Laemmli reducing buffer and incubated at 95 °C for 5 min.

### 2.10 SDS-PAGE and Western blotting

The pellet containing 5 × 10^7^ washed human spermatozoa was lysed in 100 μL of 2× concentrated Laemmli sample buffer. The 70 μL of Pierce™ RIPA buffer (ThermoFisher Scientific) was added to 50 μL sperm pellets (5 × 10^7^ cells). Both human and boar spermatozoa were lysed on ice and vortexed every 5 min for 30 min. An additional 70 μL of 2× concentrated Laemmli sample buffer was added to the boar sperm lysates in RIPA buffer. The samples from humans and boars were reduced with 5% (v/v) mercaptoethanol, boiled for 5 min, and centrifuged at 10,000×g for 2 min. Supernatants containing lysed proteins from membrane fractionation, PT isolation, and total sperm isolation were subjected to electrophoresis directly. The Mini-PROTEAN Tetra system (Bio-Rad) was used for polyacrylamide gel electrophoresis with sodium dodecyl sulfate (SDS-PAGE). Proteins were separated on a linear 12% polyacrylamide separating gel (12% (w/v) Acrylamide/Bis acrylamide solution (Bio-Rad); 1.5 M Tris-HCl (Bio-Rad), pH 8.8; 0.1% (w/v) SDS; TEMED; 0.1% (w/v) ammonium persulfate) and 4% stacking gel (4% (w/v) Acrylamide/Bis-Acrylamide solution; 0.5 M Tris-HCl, pH 6.8 (Bio-Rad); 0.1% (w/v) SDS; TEMED; 0.1% (w/v) ammonium persulfate). Precision Plus Protein Dual Color Standards (Bio-Rad) were used to estimate the molecular weights of the separated sperm proteins. After SDS-PAGE, the separated proteins were transferred onto a PVDF membrane (Millipore, USA) via electroblotting for 1.5 h at 0.5 A. The transferred proteins were visualized with Ponceau S. Subsequently, the PVDF membranes with transferred proteins were washed and blocked with 5% (w/v) Blotto non-fat dry milk (Chem Cruz, Santa Cruz Biotechnology, Inc., USA) dissolved in PBS for 1 h at RT. The membranes were then incubated with mouse monoclonal antibodies (anti-RAB2A/5C5, anti-lactadherin/1H9) diluted 1:10 in PBS at 4 °C overnight. As a negative control, membranes were co-incubated with PBS. After incubation, the membranes were washed 3× in PBS with 0.1% (v/v) Tween-20 (PBS-T) for 10 min. Subsequently, the membranes were incubated with an appropriate species-specific secondary antibody (dilution 1:3,000 in PBS) for 1 h at RT on a rolling platform. The membranes were washed 5× with PBS-T for 5 min and then once in PBS for 5 min. Next, membranes were covered with a chemiluminescent substrate (ThermoFisher Scientific); and specific protein bands, recognized by the respective antibodies, were imaged with an AZURE Biosystems C300 device (Azure Biosystems, USA). The entire uncut membranes are shown in Supplementary Figure S4. For porcine samples, equal protein loading was verified by staining the membrane with the Coomassie Brilliant Blue G-250 solution (Supplementary Figure S5Aa), and for human sperm samples, tubulin was used as a loading control (Supplementary Figure S5Ab). Control of isolated perinuclear theca was performed by H2B detection (Supplementary Figure S5).

### 2.11 Mass spectrometry analysis

Proteins from human normospermic spermatozoa were isolated according to the procedure described above in 2× concentrated Laemmli sample buffer and then loaded onto a 4%–20% gradient gel (Bio-Rad). After protein separation, the Coomassie Brilliant Blue G-250 (w/v) solution and protein bands corresponding to the size of the RAB2A protein detected on the membrane were then cut from the gel slab. The proteins were digested by trypsin. After O/N digestion at 37 °C, samples were analyzed using a liquid chromatography system Agilent 1200 (Agilent Technologies, USA) connected to the timsToF Pro PASEF mass spectrometer equipped with Captive spray (Bruker Daltonics, USA). The mass spectrometer was operated in a positive data-dependent mode. Five microliters of peptide mixture were injected by autosampler on the C18 trap column (UHPLC Fully Porous Polar C18 0.3 × 20 mm, Phenomenex, USA). After 5 min of trapping at a flow rate of 20 μL/min, peptides were eluted from the trap column and separated on a C18 column (Luna Omega 3 μm Polar C18 100 Å, 150 × 0.3 mm, Phenomenex) by a linear 35 min water-acetonitrile gradient from 5% (v/v) to 35% (v/v) acetonitrile at a flow rate of 4 μL/min. The trap and analytical columns were both heated to 50 °C. Parameters from the standard proteomics PASEF method were used to set timsTOF Pro. The target intensity per individual PASEF precursor was set to 6000, and the intensity threshold was set to 1500. The scan range was set between 0.6 and 1.6 V s/cm^2^ with a ramp time of 100 ms. The number of PASEF MS/MS scans was 10. Precursor ions in the m/z range between 100 and 1700 with charge states ≥2+ and ≤6+ were selected for fragmentation. The active exclusion was enabled for 0.4 min.

### 2.12 *In vitro* sperm-ZP binding assay–antibody block

To decipher the role of the proteins recognized by the selected antibodies, a binding assay combining motile spermatozoa with zona-intact oocytes was used (Margaryan et al., 2015). After 42–44 h of *in vitro* maturation in the NCSU-37 medium, the COCs were denuded of cumulus cells by adding 0.1% (w/v) hyaluronidase into the wells and gentle pipetting. Metaphase II (MII) oocytes were then selected based on the presence of a polar body, washed, and transferred into 500 µL of TALP medium (Piñeiro-Silva et al., 2023), and left in the incubator until the spermatozoa were added. Boar insemination doses were washed in PBS with 0.2 g/L PVA to remove the extender. To select motile spermatozoa, a swim-up procedure was performed by using NaturARTs PIG sperm swim-up medium (Embryocloud, Spain). One milliliter of sperm suspension was carefully laid beneath 1 mL of swim-up medium in a conical tube, which was then incubated at a 45° angle for 20 min at 38 °C. Subsequently, 500 µL of the upper layer containing motile spermatozoa was gently aspirated. Motile post swim-up spermatozoa were recovered and moved into TALP medium. Spermatozoa in TALP were then coincubated with in-house produced antibodies (anti-RAB2A/5C5, anti-lactadherin/1H9 diluted 1:2), hybridoma medium as vehicle control (dilution corresponding to 5C5/1H9 antibodies, diluted 1:2), and mouse IgG Isotype Control (ThermoFisher Scientific; 1 μg/mL). For the other blocking experiment, commercial anti-RAB2A antibody (#PA5-101823) in 10 μg/mL concentration and rabbit IgG Isotype Control (ThermoFisher Scientific) in the same concentration were used. Spermatozoa were incubated with the antibodies at 38.5 °C and 5% CO_2_ for 1 h while also undergoing capacitation in this medium. Treated and untreated spermatozoa were then washed in TALP, then added to MII oocytes (5 × 10^4^ spermatozoa per 10 oocytes) in TALP and co-incubated at 38.5 °C and 5% CO_2_ for 30 min. After coincubation, sperm/oocyte complexes formed via sperm adhesion were gently washed twice to remove the unbound spermatozoa and fixed in 4% (v/v) paraformaldehyde for 40 min at RT. Subsequently, samples were mounted on Teflon-coated slides in a DAPI-supplemented mounting medium (VectaShield, Vector Laboratories, Inc.). The number of bound spermatozoa per ZP-intact oocyte in all groups was counted under the epifluorescence microscope (ECLIPSE Ni, Nikon) with ×400 magnification. Sperm counts were subjected to statistical analysis.

### 2.13 *In vitro* sperm-ZP binding assay–competitive block

To validate the results from the antibody block assay, a competitive sperm-ZP binding assay (Sanz et al., 1992) was performed by using recombinant proteins: human RAB2A and pig lactadherin/MFGE8. Metaphase II oocytes and spermatozoa were processed under the same conditions as for the antibody block described above. Recombinant proteins were incubated with MII-stage ZP-intact oocytes for 60 min at a concentration of 20 μg/mL, prior to the oocyte incubation with spermatozoa (Yang et al., 2015), to competitively block the sperm binding sites on the ZP of the oocyte. Control groups included oocytes incubated with 20 μg/mL of ovalbumin (#1184201, egg albumin, lyophilized, salt-free; SERVA Electrophoresis GmbH, Germany) and oocytes incubated in TALP medium alone. Following a 30 min co-incubation with spermatozoa, the oocytes were washed, fixed, and mounted on slides with VectaShield-DAPI. The number of spermatozoa bound to each oocyte was quantified and statistically analyzed to assess the degree of inhibition of the sperm-ZP binding as described above. To confirm the attachment of the recombinant proteins to the ZP of the oocytes, the rc-RAB2A and rc-lactadherin proteins were incubated with oocytes for 60 min, washed, and incubated with the anti-RAB2A (5C5 and commercial-PA5-101823) and anti-lactadherin (1H9) primary antibodies, respectively. Negative controls were prepared by omitting the incubation with the recombinant proteins and ovalbumin. Fluorescently labelled secondary antibodies were applied, and signal localization was visualized and assessed by using epifluorescence microscopy (ECLIPSE Ni, Nikon).

### 2.14 Far Western blotting of recombinant proteins with biotinylated ZP glycoproteins

To investigate the binding affinity between the rc-RAB2A or rc-lactadherin and biotinylated ZP glycoproteins, a Far Western blot was conducted. A total of 20 µL of recombinant protein was mixed with 5 µL of Laemmli sample buffer. The proteins were then separated by using a 15% (v/v) SDS-PAGE gel and transferred onto a PVDF membrane. The membrane was then blocked with 1% (w/v) gelatin to prevent non-specific binding, and the biotinylated porcine ZP glycoproteins (Zigo et al., 2015) were diluted to a final concentration of 100 μg/mL in PBS. The membrane was incubated with the ZP glycoproteins overnight at 4 °C. After incubation, the membrane was washed, and Avidin-HRP (1 μg/mL) was applied for 1 h at RT. The membrane was visualized by using the Femto chemiluminescence substrate (ThermoFisher Scientific) and imaged with an AZURE Biosystems C600 device (Azure Biosystems).

### 2.15 Statistical analysis

Numbers of spermatozoa bound per oocyte obtained from the binding assays were normalized to the mean of the control dataset for each trial to account for inter-experimental variability. All data were expressed as mean and standard error of mean (Mean ± SEM) and results were presented as percentages. Statistical analyses for the graphs shown in the figures were conducted in GraphPad Prism 10 (v10.4.1, GraphPad Software, USA). Statistical significance between the experimental groups was determined by using the Kruskal-Wallis test, followed by Dunn’s multiple comparison test for *post hoc* comparisons. Statistical significance was set at *α* = 0.05. The densitometry analysis of Western blots was measured by the ImageJ system (Java-based image processing program, LOCI, University of Wisconsin, WI, USA), and statistical analysis was performed in BioRender.com (R version 4.2.2; Agreement number VG28BWP44I; Zigo et al., 2024) by using the Kruskal-Wallis test for comparing data between groups. The *p* value <0.05 was considered to be statistically significant.

### 2.16 Structural modeling

Protein structures of Rab2A GTPase (Eathiraj et al., 2005) and mouse IgG antibody antigen-binding fragment have been downloaded from the Protein Data Bank (PDB, DOIs: https://doi.org/10.2210/pdb1Z0A/pdb; https://doi.org/10.2210/pdb8TCA/pdb) and placed into the cell membrane model (Lomize et al., 2006) in Blender 4.4. Environment (Blender Foundation; https://www.blender.org/). Image and video demonstration of the possible differential epitope accessibility and interaction between Rab2A C-terminal domain and variable domain of IgG (5C5 antibody) in the non-permeabilized and permeabilized cells was rendered and exported using Blender Cycles render engine and animation module.

## 3 Results

### 3.1 RAB2A and Lactadherin/MFGE8 localization in boar spermatozoa at different post-testicular maturation stages and in reproductive tissue sections

To localize target sperm proteins, the anti-RAB2A (5C5) and anti-lactadherin (1H9) monoclonal mouse antibodies were employed via indirect immunofluorescence microscopy. Immunolocalization was performed on spermatozoa at different stages of their post-testicular maturation (epididymal, non-capacitated, *in vitro* capacitated, and acrosome-reacted/exocytosed), as well as on the testicular and epididymal tissue sections. The percentage of acrosome-reacted spermatozoa after the *in vitro* induction of the acrosomal exocytosis averaged 82.75% ± 3.25%.

**FIGURE 1:**
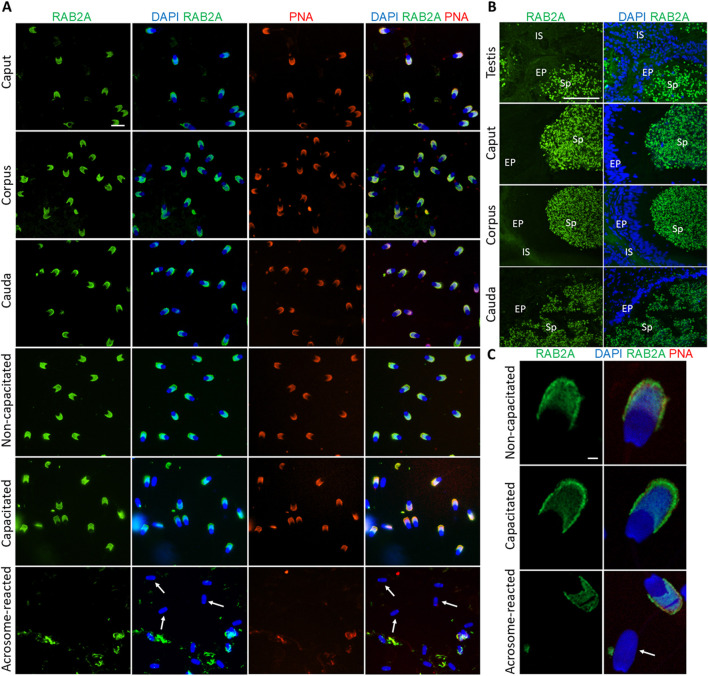
Visualization of RAB2A protein in boar spermatozoa and reproductive tissue sections. **(A)** Fluorescent signal of anti-RAB2A/5C5 antibody (green) was detected by epifluorescence microscopy in the acrosome of spermatozoa isolated from the caput, corpus, and cauda epididymis. Acrosomes were stained with lectin PNA (red), and DNA was stained with DAPI (blue). Fluorescent signal of the anti-RAB2A/5C5 antibody (green) was detected by epifluorescent microscopy in the acrosomes of non-capacitated and *in vitro* capacitated spermatozoa. No signal was detected in the spermatozoa that have undergone the acrosome reaction (white arrows). Acrosomes were stained with lectin PNA (red), and DNA was counter-stained with DAPI (blue). The scale bar represents 10 µm. Negative controls are shown in Supplementary Figure S4. **(B)** Fluorescent visualization of RAB2A in the testicular sections revealed a strong fluorescent signal in the acrosomes of sperm cells in the lumen. The signal remained strong in the acrosomes of spermatozoa in the lumen of the caput, corpus, and cauda epididymis. Sp, spermatozoa; EP, epithelial cells; IS, interstitial tissue. The scale bar represents 100 µm. Negative controls are shown in Supplementary Figures S6, S7. **(C)** High-resolution images of RAB2A localization in non-capacitated, *in vitro* capacitated, and acrosome-reacted (white arrow) spermatozoa obtained by confocal microscopy. The scale bar represents 1 µm.

The RAB2A protein was detected in the acrosomes of spermatozoa isolated from all epididymal segments, as well as in non-capacitated and *in vitro* capacitated spermatozoa. This signal disappeared after the acrosomal exocytosis, when the acrosomal cap was no longer present on the sperm head, indicating that RAB2A was not present, or detectable on the inner acrosome membrane (Figure 1A). Detection of RAB2A on the testicular tissue sections revealed a strong fluorescent signal in the acrosomes of sperm cells in the lumen of the seminiferous tubules. The signal remained strong in the acrosomes of spermatozoa visualized in the lumen of the caput, corpus, and cauda epididymal tissue sections (Figure 1B). Confocal microscopy, utilized to provide higher resolution images of non-capacitated, *in vitro* capacitated, and acrosome-reacted spermatozoa, revealed identical localization patterns of RAB2A in more detail (Figure 1C).

To assess the in-house anti-RAB2A (5C5) antibody specificity and reliability, a commercially available anti-RAB2A (PA5-101823) was used for RAB2A detection in boar spermatozoa. This antibody showed a staining pattern similar to 5C5 in permeabilized sperm (Supplementary Figure S2).

**FIGURE 2:**
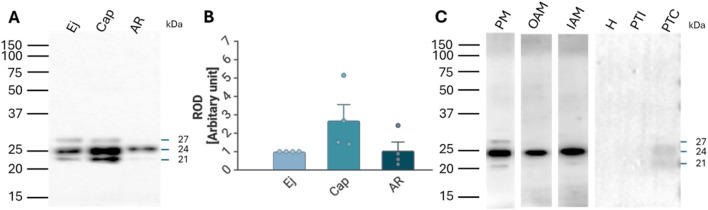
Detection and localization of RAB2A protein in boar spermatozoa. **(A)** RAB2A (5C5) antibody detected the 21, 24, and 27 kDa protein bands by Western blot analysis. All three forms were detected in the non-capacitated (ejaculated; Ej) and capacitated (Cap) spermatozoa, while the acrosome-reacted (AR) spermatozoa contained mainly the 24 kDa isoform. Each lane was loaded with protein extracted from 5 × 10^6^ sperm cells. **(B)** Densitometric analysis of the total signal did not show significant (*p* > 0.05) differences in RAB2A content between Ej, Cap, and AR spermatozoa. **(C)** Further analysis showed that the 21 and 27 kDa forms were mainly located in the sperm plasma membrane (PM) enriched fraction, while the 24 kDa isoform was present in all sperm membrane fractions, including the enriched PM, the outer acrosomal membrane (OAM), and the inner acrosomal membrane (IAM) proteins, which contain also the sperm head residual proteins. The RAB2A also contributed to the proteome of PT, namely the 21 and 24 kDa forms, which were detected in covalently-bound proteins of perinuclear theca (PTC) protein fractions. Rest of the head (H), ionically-bound proteins of the perinuclear theca (PTI). Membrane fractionation was verified by fluorescent lectin labeling of the acrosome using PNA lectin (Supplementary Figure S5B).

The presence of RAB2A protein in spermatozoa was confirmed by Western blot analysis, which detected RAB2A in non-capacitated (ejaculated; Ej) and *in vitro* capacitated (Cap) spermatozoa as the 21, 24, and 27 kDa isoforms. After the acrosome reaction, only the predominantly 24 kDa form was detected in the acrosome-reacted (AR) sperm extract containing only the retained sperm proteins (Figure 2A); however, densitometric analysis did not show significant differences in RAB2A amount between the individual sperm maturation stages (Figure 2B). Although one isoform was detected in acrosome-reacted sperm lysates, the protein was not detected in acrosome-reacted spermatozoa by immunofluorescence. This may be attributed to the isoform being retained in the perinuclear theca, which is described below, to the loss of the epitope due to the fixation, differences in processing, or to the remaining non-acrosome-reacted spermatozoa in the sample.

Fractionation of boar sperm membranes and PT isolation showed that the 21 and 27 kDa forms were mainly present in the enriched sperm plasma membrane (PM) fraction, while the 24 kDa form was detected in the fractions containing mainly the outer acrosomal membrane (OAM) with sperm tails, and inner acrosomal membrane (IAM) fraction containing also the sperm head residues, retaining the inner acrosomal membrane tightly associated with the perinuclear theca and nuclear surface, obtained after removal of the PM and OAM. In addition, 24 kDa and 21 kDa isoforms were also weakly detectable in the covalently bound proteins of PT (PTC) (Figure 2C).

**FIGURE 3:**
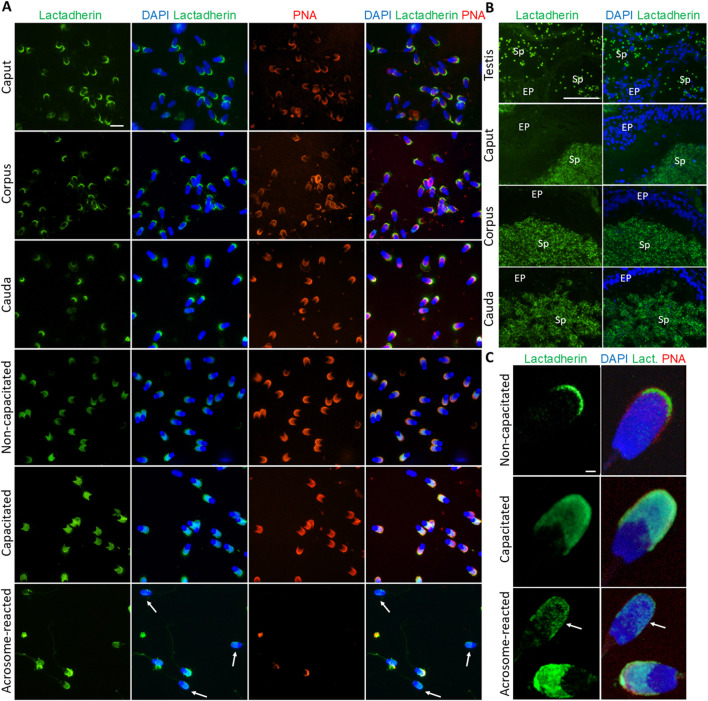
Visualization of lactadherin/MFGE8 in boar spermatozoa and reproductive tissue sections. **(A)** Fluorescent signal of anti-lactadherin antibody (1H9-green) was detected by epifluorescence microscopy in the apical ridge of the acrosome in spermatozoa isolated from the caput, corpus, and cauda epididymis. Acrosomes were stained with lectin PNA (red), and DNA was stained with DAPI (blue). Fluorescent signal of the anti-lactadherin antibody (green) was detected in the apical region of the acrosome of non-capacitated spermatozoa, and across the entire acrosome of the *in vitro* capacitated spermatozoa. A weaker signal was detected in the spermatozoa that have undergone the acrosome reaction (white arrows). Acrosomes were stained with lectin PNA (red), and DNA was stained with DAPI (blue). The scale bar represents 10 µm. Negative controls are shown in Supplementary Figure S6. **(B)** Fluorescent visualization of lactadherin/MFGE8 in the testicular section revealed a strong fluorescent signal in the apical ridge of the acrosomes in the lumen, and a weak signal in the surrounding tissue. The signal remained strong in the acrosomes of sperm cells in the lumen of the caput, corpus, and cauda epididymis. Sp, spermatozoa; EP, epithelial cells. The scale bar represents 100 µm. Negative controls are shown in Supplementary Figures S6, S7. **(C)** For better resolution, detailed images obtained by confocal microscopy display non-capacitated, *in vitro* capacitated, and acrosome-reacted (white arrow) spermatozoa. The scale bar represents 1 µm.

Fluorescent labeling of lactadherin/MFGE8, recognized by anti-lactadherin antibody (1H9), revealed its localization in the apical ridge of the acrosomes of spermatozoa isolated from the caput, corpus, and cauda epididymis. A fluorescent signal covering the entire acrosome was observed in non-capacitated spermatozoa. A bright signal localized in the apical region of the acrosome, with less intense fluorescence covering the rest of the acrosomal cap, was seen in detailed images captured by a confocal microscope. A redistribution of the signal across the entire acrosomal cap was observed in the *in vitro* capacitated boar spermatozoa. The fluorescent signal was still present in the acrosomal region after the spermatozoa had undergone the acrosomal exocytosis, indicating the target protein localization in the inner acrosomal membrane (Figure 3A). A strong fluorescent signal was present in the apical ridge of the sperm acrosomes in the lumens of the testicular seminiferous tubules. A strong signal was visible in the sperm cells in the lumens of the caput, corpus, and cauda epididymal tissues (Figure 3B). Confocal microscopy, affording higher resolution images of non-capacitated, *in vitro* capacitated, and acrosome-reacted spermatozoa, revealed the identical localization of lactadherin/MFGE8 in the apical region of the sperm head in the non-capacitated, ejaculated spermatozoa, and across the entire acrosome of the capacitated spermatozoa. A weaker signal was observed in the acrosomal region after exocytosis (Figure 3C).

**FIGURE 4:**
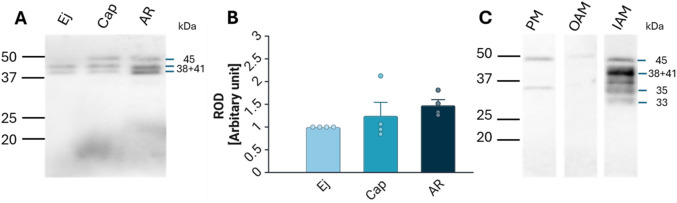
Detection and localization of lactadherin/MFGE8 in boar spermatozoa. **(A)** Antibody against lactadherin (1H9) detected 38, 41, and 45 kDa protein bands in non-capacitated (ejaculated; Ej), capacitated (Cap), and acrosome-reacted (AR) sperm extracts. Each lane was loaded with protein extracted from 5 × 10^6^ sperm cells. **(B)** Relative optical density (ROD) of the total signal did not show significant (*p* > 0.05) differences between different sperm stages. **(C)** Further analysis showed that the 45 and 35 kDa forms are located in the sperm all three membrane fractions–enriched plasma membrane (PM), outer acrosomal membrane (OAM) and inner acrosomal membrane (IAM) proteins, which contain also sperm head residual proteins, but the IAM fraction also contains additionally protein forms of 33, 38, and 41 kDa.

Lactadherin/MFGE8 was also detected by Western blot analysis as three main isoforms, at 38, 41, and 45 kDa, in Ej, Cap, and AR spermatozoa (Figure 4A), with no significant differences observed (Figure 4B). Further sperm membrane fractionation revealed that 35 and 45 kDa lactadherin/MFGE8 isoforms were present in the PM and OAM enriched fractions. The IAM fraction, containing also the sperm head residues, displayed these two isoforms, as well as the 33, 38, and 41 kDa forms of lactadherin/MFGE8 (Figure 4C).

### 3.2 Effect of antibodies and rc-RAB2A and rc-Lactadherin on sperm-ZP binding

**FIGURE 5:**
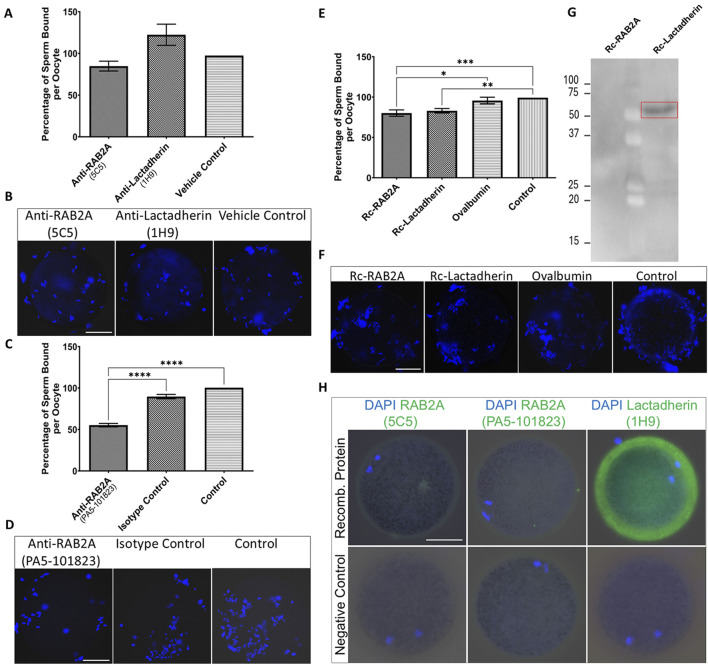
Evaluation of the number of spermatozoa bound to the ZP-intact oocytes in the *in vitro* binding assays. **(A)** The number of bound spermatozoa was non-significantly different in the anti-RAB2A (5C5, N = 40) and anti-lactadherin/MGFE8 (1H9, N = 38) groups compared to vehicle control (N = 46). **(B)** Representative images of DAPI-stained zona-bound spermatozoa in each group. **(C)** A significant reduction (***p < 0.001) in the number of zona-bound spermatozoa was observed in the commercial anti-RAB2A antibody group (PA5-101823, N = 55) compared to the isotype control (N = 51) and control without antibodies (N = 54). **(D)** Representative images of DAPI-stained zona-bound spermatozoa in each group. **(E)** A significant reduction in the number of zona-bound spermatozoa was observed in the recombinant RAB2A (N = 63) and rc-lactadherin (N = 61) groups, and no significant reduction in the ovalbumin group (N = 62), compared to the control without proteins (N = 61). **(F)** Representative images of DAPI-stained zona-bound spermatozoa in each treatment group; ***p < 0.001; **p < 0.01; *p < 0.05. **(G)** Western blot analysis of recombinant RAB2A and lactadherin/MGFE8 incubated with solubilized biotinylated ZP glycoproteins revealed a positive detection signal around 50 kDa, indicating that recombinant lactadherin/MGFE8 binds to the ZP glycoproteins. No signal was observed with the recombinant RAB2A. **(H)** Visualization of the attachment of the recombinant RAB2A to the ZP of the oocytes by using both anti-RAB2A antibodies (5C5 and PA5-101823) showed only a very weak fluorescent signal. In contrast, the anti-lactadherin antibody (1H9) showed a strong fluorescent signal in the oocytes co-incubated with rc-lactadherin. Negative controls showed no fluorescent signal. The scale bar represents 50 µm.

The presence of RAB2A protein on the surface of capacitated spermatozoa, and its implied interaction with ZP glycoproteins, raised an intriguing question about its role in the initial steps of sperm-oocyte adhesion (Zigo et al., 2015). Similarly, while multiple physiological functions of lactadherin/MFGE8 have been proposed, its role in porcine fertilization remains unresolved, warranting the investigation of its affinity towards the ZP glycoproteins. To follow up on the hypothesis that RAB2A and lactadherin/MFGE8 are involved in the sperm ZP-binding in the pig, the *in vitro* sperm-zona binding assays were conducted. Spermatozoa pretreated with anti-RAB2A or anti-lactadherin antibodies were co-incubated with ZP-intact oocytes, and the number of spermatozoa bound to each oocyte in all groups was quantified using fluorescence microscopy. Statistical analysis of the datasets obtained from these binding assays revealed no significant (*p* > 0.05) reduction in oocyte binding in both anti-RAB2A (N = 40) and anti-lactadherin antibody group (N = 38), compared to a vehicle control (N = 46) represented by the hybridoma medium (Figures 5A,B), in which the antibodies were prepared and stored. A Kruskal-Wallis H test revealed no statistically significant difference in sperm binding between the three groups, *H(2)* = 5.66, *p* = 0.059. *Post hoc* analysis using Dunn’s multiple comparisons test showed no significant differences between anti-RAB2A (5C5) and vehicle control (*mean rank difference (MRD)* = 9.75, *z* = 1.25, *p* = 0.419), nor between anti-lactadherin (1H9) and vehicle control (*MRD* = 9.62, *z* = 1.22, *p* = 0.444). Due to the similarly insignificant inhibition of sperm-zona binding observed in the vehicle control and in both the anti-RAB2A and anti-lactadherin antibody groups (Supplementary Figure S8), concerns were raised about potential non-specific effects of the vehicle. To address this issue, a commercially available, highly concentrated anti-RAB2A antibody was used in follow-up experiments to minimize vehicle-related interference. These additional experiments revealed a significant inhibition of sperm-zona binding in the anti-RAB2A (PA5-101823, N = 55) compared to both the isotype (N = 51) and vehicle control (N = 54) (Figures 5C,D). A Kruskal-Wallis H test showed a statistically significant difference between groups, *H(2)* = 81.14, p < 0.001. *Post hoc* analysis using Dunn’s multiple comparisons test showed significant differences between anti-RAB2A and isotype control (*MRD* = −59.56, *z* = 6.61, *p* < 0.001), and between anti-RAB2A and control (*MRD* = −76.03, *z* = 8.57, *p* < 0.001). No significant difference was observed between the isotype control and control (*MRD* = −16.48, *z* = 1.82, *p* = 0.206).

To further evaluate the role of candidate proteins in the ZP binding, a competitive binding assay was conducted by using commercially available rc-RAB2A and rc-lactadherin. Ovalbumin was used as a control of steric hindrance based on its molecular weight (45 kDa) corresponding to the molecular weight of the 6xHis-GST tagged rc-RAB2A (human; 52.4 kDa), and 6xHis-tagged rc-lactadherin (porcine; 52 kDa). Individual recombinant proteins or ovalbumin were coincubated with ZP-intact MII oocytes prior to the coincubation with spermatozoa, and a control group of non-treated oocytes incubated with spermatozoa in TALP medium was included as well. The number of spermatozoa bound to the oocytes was counted and statistically analyzed. Both groups containing recombinant proteins (rc-RAB2A N = 63; rc-lactadherin N = 61) exhibited a weak, yet significant reduction in the number of bound spermatozoa compared to the control (N = 61) (Figures 5E,F). A Kruskal-Wallis H test indicated a significant difference between the four groups, *H(3)* = 17.86, *p* < 0.001. Dunn’s multiple comparisons test showed that rc-RAB2A had a significantly higher mean rank than ovalbumin (*MRD* = 33.02, *p* = 0.049) and control (*MRD* = 45.77, *p* = 0.002). The rc-lactadherin also had a significantly higher mean rank than control (*MRD* = 41.48, *p* = 0.007). Differences between rc-lactadherin and ovalbumin (*MRD* = 28.73, *p* = 0.129) and between ovalbumin and control (*MRD* = 12.74, *p* > 0.999) were not statistically significant.

To examine the binding affinity of the recombinant proteins to ZP glycoproteins, a Far Western blot binding assay of rc-RAB2A and rc-lactadherin incubated with solubilized biotinylated porcine ZP glycoproteins was carried out. The interaction was detected around 50 kDa, indicating that rc-lactadherin binds to the ZP glycoproteins (Figure 5G). No interaction was observed with the rc-RAB2A. We believe that the lack of interaction may be caused by conformational changes or partial denaturation of the human rc-RAB2A and the denatured state of the porcine ZP glycoproteins in the Far Western blot binding assay. Nevertheless, human RAB2A is highly conserved with porcine RAB2A (Supplementary Figure S9), indicating that species differences are unlikely to explain the absence of binding. To further verify the binding of the recombinant proteins to the ZP-intact oocytes, their detection was performed after incubation with MII oocytes by using both commercial and in-house antibodies to RAB2A and lactadherin/MFGE8. Negative controls were prepared by omitting the recombinant proteins from the coincubation. Visualization of the attached rc-RAB2A to the ZP of the oocytes by using 5C5, and commercial anti-RAB2A antibody showed only a very weak to no fluorescent signal, while the bound rc-lactadherin showed a strong fluorescent signal on the zona (Figure 5H). The absence of rc-RAB2A detection does not necessarily imply that the recombinant protein does not attach to the ZP, as several factors, such as GST-tag induced steric hindrance or close proximity of the ZP-interacting domain and antibody epitope, could contribute to this lack of detection.

### 3.3 Detection of RAB2A and Lactadherin/MFGE8 in its native form in non-permeabilized spermatozoa

To assess the presence of RAB2A and lactadherin/MFGE8 on the surface of live boar spermatozoa, immunolabeling was performed on sperm samples in suspension without prior fixation or permeabilization, aiming to corroborate the participation of these proteins in the sperm-ZP interaction.

**FIGURE 6:**
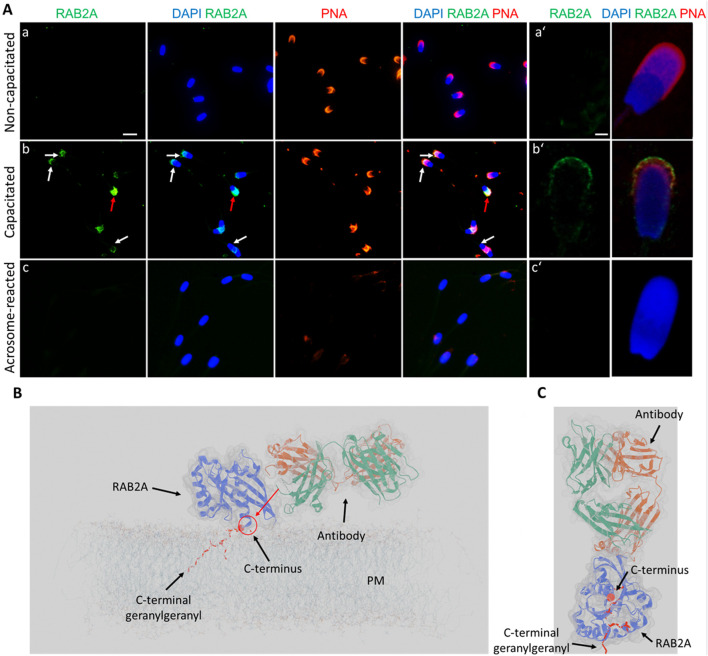
Visualization of RAB2A in non-permeabilized boar spermatozoa. **(A)** Fluorescent signal of the anti-RAB2A antibody (5C5-green) was not detected in the non-capacitated spermatozoa prepared in suspension; however, it was detectable in the apical acrosomal region of capacitated spermatozoa (white arrows). The red arrow indicates a stronger signal in the entire acrosome of a damaged sperm cell. No signal was detected in spermatozoa undergoing the acrosome reaction. Acrosomes were counter-stained with lectin PNA (red) and DNA with DAPI (blue). The scale bar represents 10 µm. **(B)** High-resolution images were obtained by confocal microscopy. The scale bar represents 1 µm. **(C)** Modeling of the differential epitope accessibility and interaction between the C-terminal domain of RAB2A and anti-RAB2A antibody. The red arrow indicates the inaccessibility of a potential epitope for antibody binding. **(D)** Modeling of the antibody orientation toward RAB2A. The C-terminal domain is indicated by the red sphere, and the lipid anchor by the red ribbon. A corresponding 3D video is available as Supplementary Video file. Negative controls are shown in Supplementary Figure S11.

In the case of RAB2A, the non-permeabilized, non-capacitated spermatozoa exhibited no detectable fluorescent signal, except for a few cells that appeared not fully intact. *In vitro* capacitated spermatozoa displayed a visible fluorescent signal in the apical region of the acrosomes. After the induction of acrosome reaction, no signal was observed in acrosome-reacted spermatozoa, reinforcing the finding that RAB2A is shed by exocytosis (Figures 6A,B). During ongoing capacitation, RAB2A detection likely reflects membrane reorganization and its redistribution into hybrid vesicles at the initial stage of acrosomal exocytosis (Supplementary Figure S10). We hypothesize that the epitope recognized by the antibody is close to the C-terminal anchoring of the protein to the intact, i.e., the non-capacitated or chemically permeabilized plasma membrane. This is supported by immunofluorescence labeling results on mouse spermatozoa, which showed no signal following incubation with the anti-RAB2A antibody. Notably, the mouse RAB2A protein has a difference in its amino acid sequence in this region (Supplementary Figure S9). The predictive model suggested that in non-permeabilized cells, the accessibility of RAB2A C-terminal epitope to 5C5 antibody recognition is limited (Figures 6C,D). This also proposed the effect of membrane permeabilization or membrane increased fluidity and reorganization during capacitation could further enhance epitope accessibility. The side view (Figure 6C) and top view (Figure 6D) show selected perspectives, while the Supplementary Video presents the overall concept.

The anti-RAB2A (PA5-101823) antibody showed the same fluorescent signal as the 5C5 antibody in capacitated spermatozoa in unpermeabilized sperm samples (Figure 1), which disappeared after the acrosome exocytosis (Supplementary Figure S2). In addition, a very weak signal was observed in acrosomal area of non-capacitated spermatozoa incubated in suspension with the commercial antibody (Supplementary Figure S2).

**FIGURE 7:**
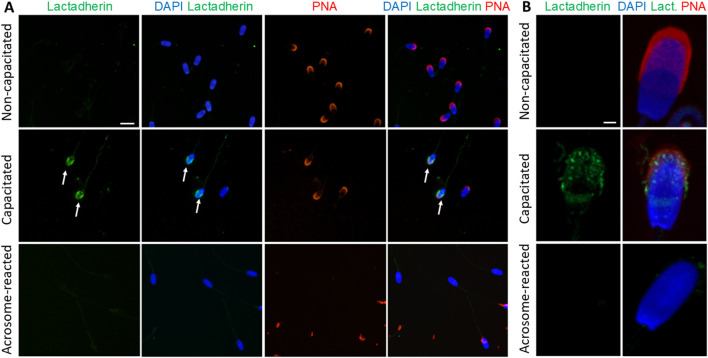
Visualization of lactadherin/MFGE8 in non-permeabilized boar spermatozoa. **(A)** Fluorescent signal of the anti-lactadherin antibody (1H9-green) was not detected in the non-capacitated spermatozoa, while it was prominent in the acrosomes of capacitated spermatozoa (white arrows). No visible signal was observed in the acrosome-reacted spermatozoa. Acrosomes were counter-stained with lectin PNA (red) and DNA with DAPI (blue). The scale bar represents 10 µm. **(B)** High-resolution images were obtained by confocal microscopy. The scale bar represents 1 µm. Negative controls are shown in Supplementary Figure S11.

Similarly, lactadherin/MGFE8 was not detected in the non-permeabilized, non-capacitated spermatozoa prepared in suspension, but a robust signal was shown in the acrosomes of capacitated spermatozoa. No visible signal was observed in spermatozoa that had undergone acrosomal exocytosis (Figures 7A,B).

### 3.4 Visualization and detection of RAB2A in human spermatozoa

**FIGURE 8:**
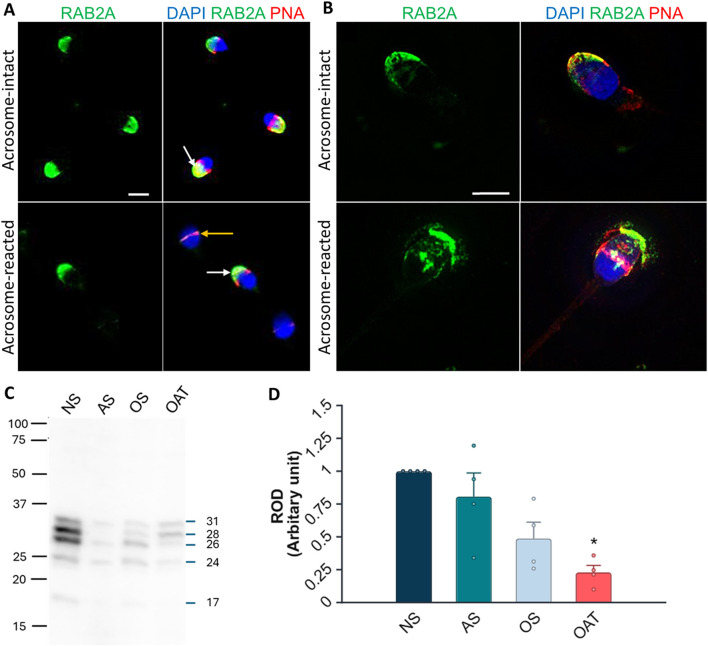
Localization and detection of RAB2A in human spermatozoa. **(A)** Fluorescent signal of the anti-RAB2A antibody (5C5-green) was detected by epifluorescent microscopy in the acrosome-intact spermatozoa fixed with paraformaldehyde (white arrow); however, the fluorescent signal was absent from the apical region of acrosome-reacted spermatozoa (yellow arrow). **(B)** Fluorescent signal of the anti-RAB2A was also detected by SIM in acrosome-intact human spermatozoa, and disrupted by acrosomal exocytosis (red), causing the loosening/release of RAB2A from spermatozoa (green). The presence of acrosomes was confirmed with lectin PNA (red), and DNA was counter-stained with DAPI/Hoechst 33342 (blue). The scale bar represents 3 µm. **(C)** The RAB2A protein (31, 28, 26, 24, 17 kDa) was detected in the normozoospermic (NS), asthenozoospermic (AS), oligozoospermic (OS), and oligoasthenoteratozoospermic (OAT) sperm lysates. Each lane was loaded with protein extracted from 5 × 10^6^ sperm cells. **(D)** Relative optical density (ROD) confirmed a decreased RAB2A protein content in spermatozoa from men with specific etiologies of male infertility (N = 4), with a significant reduction observed in OAT samples in comparison to normospermic samples (**p < 0.01).

We were interested in whether our 1H9 and 5C5 antibodies cross-react with target proteins in human spermatozoa. In the case of the 1H9 antibody, we obtained negative results by both immunofluorescence and Western blotting (Supplementary Figure S12); though, the 5C5 antibody detected RAB2A protein in human spermatozoa. By immunofluorescence labeling, we detected a signal in the acrosomal region of acrosome-intact spermatozoa (Figure 8A), which disappeared after acrosomal exocytosis. By using SIM, we were able to detect the release of RAB2A protein within the exocytosing acrosome (Figure 8A). The presence of RAB2A protein was also confirmed by Western blotting, detecting the 31, 28, 26, and 24 kDa forms in ejaculated human spermatozoa (Figure 8B). Mass spectrometry analysis of normospermic samples confirmed the presence of RAB2A protein at multiple molecular weights (blue arrows in Supplementary Figure S13A). Detected peptides covered at least 55% of the protein sequence for the least-covered protein and up to 76% for the most-covered protein. Peptides are highlighted in gray and underlined with a blue line, while post-translational modifications, including acetylation, carboxymethylation, or oxidation, are indicated in color on the corresponding amino acids in Supplementary Figure S13B. Since RAB2A is crucial for acrosome biogenesis (Mountjoy et al., 2008), and we observed its release during the acrosomal exocytosis, we were interested in whether RAB2A could be used as a biomarker of sperm quality. We detected all four isoforms of RAB2A with the 5C5 antibody in Western blot analysis of spermatozoa from NS, AS, OS, and OAT men (N = 4; Figure 8B). The densitometric analysis revealed a relative decrease in total RAB2A protein in pathological sperm samples. A Kruskal-Wallis test revealed a significant difference among the groups, *H(3) =* 8.49*, p =* 0.037. *Post hoc* comparisons using Dunn’s test indicated that the NS group differed significantly from the OAT group (*p =* 0.018). In contrast, differences between NS vs. AS (*p >* 0.999) and NS vs. OS (*p =* 0.475) were not significant. Descriptive statistics for each group were as follows (mean ± SEM): NS: 3.04 ± 0.83, AS: 1.57 ± 0.31, OS: 1.05 ± 0.21, OAT: 0.53 ± 0.20 (Figure 8D).

## 4 Discussion

The Ras-related protein RAB2A and lactadherin/MFGE8 (p47/SED1) have been proposed as candidate receptors mediating primary sperm binding to the ZP in the domestic pig (Ensslin et al., 1998; van Gestel et al., 2007; Zigo et al., 2015). Among the proteins identified on the boar sperm surface, RAB2A stands out as a promising but understudied candidate. As a member of the RAB family of small GTPases in the Ras superfamily, RAB2A undergoes prenylation, the addition of geranylgeranyl groups to C-terminal cysteines, which is essential for membrane anchoring (Pereira-Leal and Seabra, 2000; 2001; Gomes et al., 2003). RAB proteins play a role in vesicular transport, including vesicle formation, cytoskeletal trafficking, and membrane fusion, and have been identified across various species in multiple cell types and tissues, where they perform a wide range of diverse functions (Stenmark and Olkkonen, 2001; Liu and Storrie, 2012; Goud et al., 2018). They have been previously detected in the bovine, mouse, porcine, and human spermatozoa (Mountjoy et al., 2008; Bustos et al., 2012; Zigo et al., 2015; Bae et al., 2019).

The exact role and mechanism of RAB proteins’ function in spermatozoa have not yet been fully established, although their involvement in capacitation and acrosome reaction, as well as sperm motility regulation is implied, and may potentially serve as biomarkers of male fertility (Kwon et al., 2015; Bae et al., 2019; Bae et al., 2022). RAB2A has been implicated in the transport and fusion of small secretory vesicles from the Golgi apparatus into growing pro-acrosome and acrosome vesicles, to ensure their fusion during acrosomal biogenesis in bovine spermatids. After the completion of spermiogenesis, RAB2A remains packaged in a protein complex anchored to the subacrosomal layer of the perinuclear theca, a structure important for stabilizing the acrosome (Mountjoy et al., 2008; Oko and Sutovsky, 2009). Recently, RAB2A has been reported in the perinuclear theca of the boar spermatozoa as well (Zhang et al., 2022). This finding has been supported by the present study, where protein bands of 21 and 24 kDa were faintly detectable in covalently bound perinuclear theca protein fractions by Western blot analysis.

In mice, Bae et al. (2019) observed RAB2A in the connecting piece and the rest of the flagellum. Mountjoy et al. (2008) localized RAB2A to the cytosolic face of the acrosomal membranes of bull spermatids during spermatogenesis, suggesting an intracellular localization. Contrastly, Zigo et al. (2015) located RAB2A on the boar sperm surface which led them to the proposal of a functional role of RAB2A in the sperm-ZP binding. They detected RAB2A in protein extract isolated from the surface of *in vitro* capacitated boar spermatozoa, in which RAB2A was detected in two isoforms, 24 kDa and 27 kDa. The existence of two isoforms of this protein was explained by the hypervariability of the C-terminal domain. Using immunofluorescence, RAB2A presented the strongest signal on capacitated spermatozoa, and both isoforms were assumed to interact with ZP glycoproteins (Zigo et al., 2015). The detection of multiple different forms of RAB2A in sperm across various studies (Zigo et al., 2015; Zigo et al., 2024), including our present one, can be explained by differences in detection sensitivity, lysis and separation conditions, as well as possible individual variability, for example within a boar breed.

In the present study, three major RAB2A isoforms (21, 24, and 27 kDa) were identified in non-capacitated, capacitated, and acrosome-reacted spermatozoa. Similar calculated molecular weights have been reported for porcine RAB2A in the UniProtKB database (https://www.uniprot.org; UniProt IDs: A0A8D1Z2A2, A0A480X841, F1RT87). The 21 and 27 kDa isoforms were detected in the sperm PM protein enriched fraction, while the 24 kDa isoform was also detected in IAM and OAM protein-enriched fractions. We hypothesize that during the immunofluorescence staining of spermatozoa, the inner acrosomal membrane (IAM) may not be permissive to the antibodies, which prevents them from reaching the PT-enclosed epitope, or the density of PT itself may shroud the epitope, and therefore, no fluorescent signal is detected after acrosomal exocytosis. Despite previous reports of decreased RAB2A abundance post-capacitation (Kwon et al., 2014; Bae et al., 2019), we found no significant changes in total abundance across sperm maturation stages. While the 24 kDa isoform was present in all sperm maturation stages, the 27 kDa isoform was detected only in the ejaculated and capacitated spermatozoa, and not in the spermatozoa after the induction of the acrosomal exocytosis, suggesting that it may participate in the sperm-ZP binding and then get shed during the exocytosis. In human spermatozoa, four major isoforms (24, 26, 28, and 31 kDa) were detected, along with a minor 17 kDa isoform. In AS, OS, and especially OAT, we detected the identical RAB2A isoforms as in normozoospermic samples; however, densitometry revealed a significant decrease in the total RAB2A in OAT pathology. If RAB2A indeed drives acrosomal biogenesis, its lower abundance in spermatozoa with a greater incidence of morphological defects (acrosome-less spermatozoa in particular) is unsurprising, making RAB2A a promising candidate biomarker of acrosomal integrity and overall sperm quality.

In line with previous study (Zigo et al., 2015), by using immunofluorescence microscopy, we localized RAB2A in boar spermatozoa isolated from all segments of the epididymis as well as in the epididymal sections. However, in contrast to the previous assumptions that RAB2A is delivered to the sperm surface via epididymosomes during epididymal sperm transit (Zigo et al., 2015), we did not observe RAB2A signal in secretory epididymal tissue which is a source of extracellular vesicles and the signal was already present in testicular spermatozoa in the testicular seminiferous tubule lumens. This finding aligns with its role in acrosomal vesicle transport from the trans-Golgi during spermatid differentiation (Mountjoy et al., 2008; Morohoshi et al., 2021). Consistent acrosomal localization of RAB2A was observed across different post-testicular maturation stages. The immunofluorescent signal disappeared following the acrosome reaction, suggesting the absence of RAB2A from the inner acrosomal membrane and its shedding from the outer membrane during exocytosis. Membrane fractionation and Western blot analysis revealed that the 24 kDa isoform was retained in the enriched fraction containing IAM proteins along with sperm head residues, indicating that part of the protein persists intracellularly after acrosomal exocytosis. This apparent discrepancy may be attributed to the inaccessibility of the epitope in the intact spermatozoa due to its compartmentalization, highlighting the importance of combining immunolocalization techniques with biochemical fractionation.

Mountjoy et al. (2008) found RAB2A to be present on the cytosolic side of the forming acrosome in bovine spermatids. In contrast, Zigo et al. (2015) reported its presence on the boar sperm surface. Interestingly, our results demonstrated a limited detectability in the non-permeabilized, non-capacitated sperm samples, but showed a fluorescence signal in capacitated spermatozoa. However, the punctate pattern of the labeling suggested that RAB2A may be associated with forming hybrid PM/OAM vesicles rather than with the sperm surface. While the mechanism underlying the apparent relocalization or surface exposure of a cytosolic protein remains unclear, it may involve capacitation-associated membrane remodeling or membrane disruptions that transiently expose intracellular domains, since relocalization of intra-acrosomal proteins, such as acrosin and acrosin binding protein (ACRBP), to the sperm surface has been described in capacitating boar spermatozoa (Kongmanas et al., 2015). Notably, our recent study using the same anti-RAB2A (5C5) antibody reported a reduced signal after capacitation (Zigo et al., 2024), which may reflect differences in epitope accessibility due to fixation conditions rather than true changes in protein localization. Antibodies serve as an essential tool for the accurate detection, localization, and functional characterization of candidate gamete receptors; however, their performance can vary depending on species-specific antigen properties. While commercial antibodies for RAB2A and lactadherin/MFGE8 are available, they are typically raised against human or murine antigens. We believe that our monoclonal antibody 5C5 recognizes an epitope near the C-terminal geranylgeranyl anchor, showing specificity for porcine and human RAB2A but not for mouse orthologs, likely due to a minor sequence divergence in the C-terminus. This is partly supported by the lack of detection in mouse spermatozoa despite confirmed RAB2A expression in this species, where the protein has been shown to participate in acrosomal biogenesis (Morohoshi et al., 2021). Molecular scheme derived from crystal structures suggested epitope accessibility between the RAB2A C-terminal domain and the immunoglobulin variable domain under varied conditions. The epitope accessibility considerably increases after permeabilization of the sperm membrane or after sperm capacitation, when the membrane fluidity changes.

The 5C5 antibody did not specifically reduce the numbers of zona-bound spermatozoa after sperm-antibody preincubation. In contrast, the commercial antibody significantly reduced sperm-oocyte binding, which may reflect differences in epitope recognition, and supporting RAB2A role in the binding. Our competitive binding assay using recombinant human GST-conjugated RAB2A protein showed a weak, yet significant reduction in sperm-oocyte binding. Binding of rc-RAB2A to the oocyte was also tested, but the protein was detected only with very low intensity in its native state by both in-house 5C5 and the commercial antibody. The GST tag, present in rc-RAB2A but absent from rc-lactadherin, could sterically hinder antibody binding during the competitive sperm-ZP binding assay (Harper and Speicher, 2008). The failure of the immunodetection of rc-RAB2A bound to the ZP may also imply that the ZP-interacting domain is too close to the antibody-binding epitope However, the specific role of RAB2A during the initial contact with the ZP versus its involvement in later membrane remodeling events associated with the onset of the acrosomal exocytosis remains unclear.

Lactadherin/MFGE8 (p47/SED1), a peripheral membrane protein, was identified as a promising sperm-ZP binding candidate in boar spermatozoa by using affinity chromatography on immobilized ZP glycoproteins and Far Western blotting with biotinylated ZP (Ensslin et al., 1995; van Gestel et al., 2007; Zigo et al., 2015). Its mouse homolog MFGE8/SED1 has been shown to mediate sperm-ZP binding and considered essential for fertilization, as SED1-deficient males are subfertile (Ensslin and Shur, 2003). In pigs, lactadherin/MFGE8 has been localized to the acrosomal region of testicular, epididymal, non-capacitated, and *in vitro* capacitated spermatozoa, with changes in expression and distribution during epididymal transit and the capacitation process (Ensslin et al., 1998; Petrunkina et al., 2003; Tanphaichitr et al., 2015; Zigo et al., 2015). Lactadherin/MFGE8 is also known to interact with sulfated Lewis X structures and integrins in the oviductal epithelium, suggesting a role in sperm reservoir formation (Petrunkina et al., 2003; Silva et al., 2017).

In the present study, immunohistochemistry and fluorescence microscopy revealed lactadherin/MFGE8 in boar spermatozoa samples throughout the reproductive tract, primarily localized to the apical ridge of the acrosomal cap. No redistribution was observed during epididymal maturation. However, in ejaculated and *in vitro* capacitated spermatozoa, lactadherin/MFGE8 progressively spread across the entire acrosomal surface, as documented by confocal microscopy. In the acrosome-reacted spermatozoa, a weaker signal remained on the inner acrosomal membrane, indicating its retention after the vesiculation of the outer acrosomal membrane and formation of the acrosomal shroud. Lactadherin/MFGE8 was undetectable on the surface of non-permeabilized, non-capacitated spermatozoa, likely due to epitope masking. Nevertheless, it became accessible to the antibody on capacitated spermatozoa, aligning with previous observations (Zigo et al., 2015).

Similar to RAB2A, Western blot analysis of lactadherin/MFGE8 identified three isoforms (38, 41, and 45 kDa) in whole sperm lysates. Previous studies reported variable isoform patterns, including 37 and 47 kDa (Silva et al., 2017), 48 and 33 kDa (Zigo et al., 2019), or a single 45–47 kDa band (Ensslin et al., 1998; Petrunkina et al., 2003). Kongmanas et al. (2015) further identified this protein in the isolated boar sperm apical plasma membrane samples as the mature form of 47 kDa as well as a 39 kDa isoform, which they described as possibly a degraded or processed product. In *Sus scrofa*, UniProtKB database (https://www.uniprot.org) describes lactadherin (MFGE8) as a ∼45.7 kDa protein with seven computationally mapped potential isoforms ranging from predicted molecular weights of 38–54.5 kDa. As observed for RAB2A protein, the presence of various forms of lactadherin/MFGE8 in sperm described in different studies, including our own, may reflect variations in detection sensitivity, sample preparation, separation methods, and potential individual differences.

These lactadherin/MFGE8 isoforms were found across the enriched protein membrane fractions. While 35 and 45 kDa isoforms were detectable in the PM-enriched fraction, multiple isoforms (33, 35, 38, 41, and 45 kDa) were detected in the IAM-enriched fraction, which we attribute to the increased concentration and enrichment of these isoforms relative to whole sperm lysates, allowing for improved detection sensitivity. Thus 33 kDa isoform could be suggesting that it may be more tightly associated or under-represented in whole-cell lysates due to detergent resistance.

Lactadherin/MFGE8 has been shown to interact with proteasomal subunits, suggesting its potential degradation by the ubiquitin-proteasome system (UPS) during fertilization (Miles et al., 2013). This is supported by evidence that the UPS may mediate lactadherin/MFGE8 redistribution and degradation during capacitation (Zigo et al., 2019). However, its continued presence in capacitated spermatozoa supports its proposed role as a ZP-binding receptor (Petrunkina et al., 2003; van Gestel et al., 2007; Zigo et al., 2015). Binding assays were performed to assess the potential role of lactadherin/MFGE8 in the sperm-ZP interaction. Although anti-lactadherin antibody-treated spermatozoa showed reduced ZP binding, this inhibition was not significant compared to the hybridoma medium control. However, rc-lactadherin reduced sperm binding to the ZP in a weak, yet significant manner, compared to the control, while ovalbumin, a protein of comparable molecular weight, did not affect the number of spermatozoa bound to oocytes. This supports the lactadherin-mediated inhibition, similarly to the effect observed with rc-RAB2A, although the limitations of this approach, such that recombinant antigens may not accurately mimic the natural binding conformation of native proteins, or that the absence of native post-translational modifications or membrane environments can influence binding activity, must be acknowledged. However, Western blot analysis confirmed that rc-lactadherin binds solubilized, biotinylated ZP glycoproteins, and immunodetection demonstrated its attachment to the ZP of intact oocytes. These results support the potential involvement of lactadherin/MFGE8 in sperm-ZP interaction in pigs. However, the possibility of its synergy with other surface proteins, especially given its presence in high-molecular-weight protein complexes isolated from anterior head plasma membrane vesicles in boar sperm (Kongmanas et al., 2015), remains to be investigated. Future identification of the binding partners of both RAB2A and lactadherin/MFGE8 could contribute to a more detailed understanding of the molecular mechanisms underlying sperm-oocyte recognition.

## 5 Conclusion

The present study provided further validation of the monoclonal antibodies 5C5 and 1H9 as probes suitable for phenotyping of RAB2A and lactadherin/MFGE8 in boar, respectively, human spermatozoa. Both antibodies proved to be target-specific, selectively recognizing sperm-specific isoforms of RAB2A and lactadherin/MFGE8, respectively. Immunofluorescence analysis revealed that RAB2A and lactadherin/MFGE8 become detectable on the sperm surface upon capacitation, whereas in non-capacitated spermatozoa, the absence of the signal likely reflects epitope inaccessibility or signal levels below the microscopic detection threshold. Importantly, the detection of proteins in non-permeabilized, capacitated spermatozoa confirms the exposure of both RAB2A and lactadherin/MFGE8 on the sperm surface at the time of the initial sperm-oocyte interaction. Considering their subcellular localization to acrosomal membranes, these proteins may also participate in subsequent events related to ongoing or completed acrosomal exocytosis. Our results reinforce the concept that RAB2A plays multiple roles in sperm development and function. It contributes to acrosomal biogenesis, is retained in the perinuclear theca, and is redistributed during capacitation, potentially exposing the binding domains for sperm-ZP recognition. By using the 5C5 antibody, we detected reduced RAB2A levels in samples from men with pathological spermiograms, qualifying RAB2A for a candidate biomarker of sperm quality and acrosomal integrity. Compared to a commercially available RAB2A antibody, the 5C5 antibody exhibits higher specificity, recognizing multiple isoforms of RAB2A present in spermatozoa, highlighting its potential as a valuable tool for assessing sperm function, acrosomal integrity, and male fertility. While in-house antibody-mediated inhibition of sperm-ZP binding was not significant, recombinant proteins significantly reduced this interaction, supporting the previously established role of lactadherin/MFGE8 and RAB2A in the sperm-ZP binding, as well as indicating that RAB2A also may contribute to sperm-ZP binding in pigs. Given the ability of commercial RAB2A antibody to significantly inhibit sperm-oocyte binding *in vitro*, further investigation into epitope-specific interactions is warranted to establish RAB2A as a ZP-binding receptor. Together, these data support a potential role for both proteins, possibly in cooperation with other sperm surface proteins, although further investigation is needed to clarify their contributions. Future studies should also focus on identifying the interacting partners of RAB2A and lactadherin/MFGE8, and elucidating their potential involvement in signaling pathways leading to the acrosome reaction.

## In Memoriam

Dedicated to the memory of our dear colleague, Assoc. Prof. Vera Jonakova, DSc.

## Data Availability

The original contributions presented in the study are included in the article/Supplementary Material, further inquiries can be directed to the corresponding author.
