# Supplementary material for: Functional characterization and phenotyping of RAB2A and Lactadherin/MFGE8 as boar sperm zona pellucida binding proteins
Source: Front Cell Dev Biol. 2025 Oct 2;13:1653053. doi: 10.3389/fcell.2025.1653053 (PMC12528150; doi:10.3389/fcell.2025.1653053)
Supplement: Supplementary file 1 [file DataSheet1.pdf]

Functional Characterization and Phenotyping of RAB2A and Lactadherin/MFGE8 as Boar Sperm Zona Pellucida Binding Proteins

Natalie Zelenkova[1], Veronika Kraus[2], Lukas Ded[2], Daniela Spevakova[2], Lucie Sadilkova[2], Michaela Frolikova[2], Aneta Pilsova[1], Zuzana Pilsova[1], Barbora Klusackova[1], Ondrej Simonik[2], Eva Chmelikova[1], Tereza Krejcova[1], Michal Zigo[3], Peter Sutovsky[3,4], Marketa Sedmikova[1], Katerina Komrskova[2,5], Pavla Postlerova[1,2*]

[1]Department of Veterinary Sciences, Faculty of Agrobiology, Food and Natural Resources, Czech University of Life Sciences, Prague, Czech Republic

[2]Laboratory of Reproductive Biology, Institute of Biotechnology of the Czech Academy Sciences, BIOCEV, Vestec, Czech Republic

[3]Division of Animal Sciences, College of Agriculture, Food and Natural Resources, University of Missouri, Columbia, MO, USA

[4]Department of Obstetrics, Gynaecology & Women's Health, University of Missouri, Columbia, MO, USA

[5]Department of Zoology, Faculty of Science, Charles University, Prague, Czech Republic

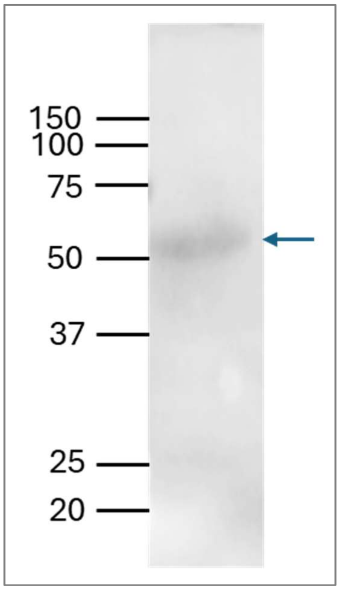

SUPPLEMENTARY FIGURE S1 | Verification of the 1H9 antibody specificity by Western blot analysis using recombinant lactadherin. Western blot analysis demonstrated the ability of 1H9 antibody to detect recombinant lactadherin. The specific signal is indicated by the blue arrow.

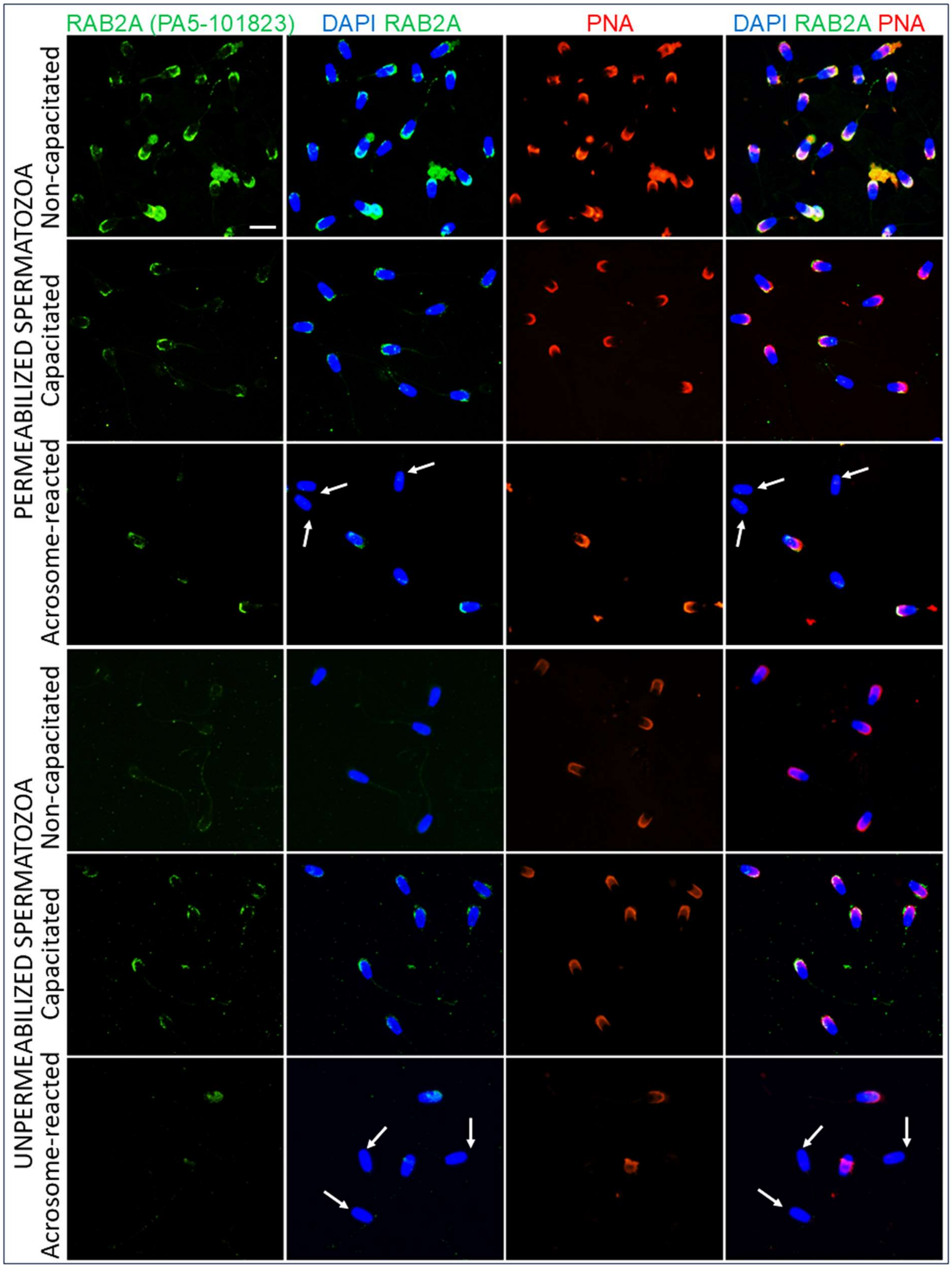

SUPPLEMENTARY FIGURE S2 | Verification of in-house anti-RAB2A antibody (5C5) specificity by immunofluorescence using commercial anti-RAB2A antibody in boar spermatozoa. To assess the in-house anti-RAB2A antibody specificity and reliability, a commercially available anti-RAB2A (PA5-101823) was used in conjunction with fluorescence microscopy in both permeabilized and unpermeabilized sperm samples during sperm post-testicular maturation. The commercial anti-RAB2A antibody (green) labeled the acrosomes of non-capacitated and *in vitro* capacitated sperm, and no signal was observed on the acrosome-reacted sperm in permeabilized spermatozoa (white arrows), in line with the results obtained by the in-house anti-RAB2A. In unpermeabilized sperm samples, a weak fluorescent signal (green) was detected in non-capacitated spermatozoa, and a visible signal was observed in in vitro capacitated spermatozoa, which disappeared after the acrosome exocytosis (white arrows). Acrosomes were stained with lectin PNA (red), and DNA was stained with DAPI (blue). The scale bar represents 10 µm. Negative controls are shown in Supplementary Figure S3.

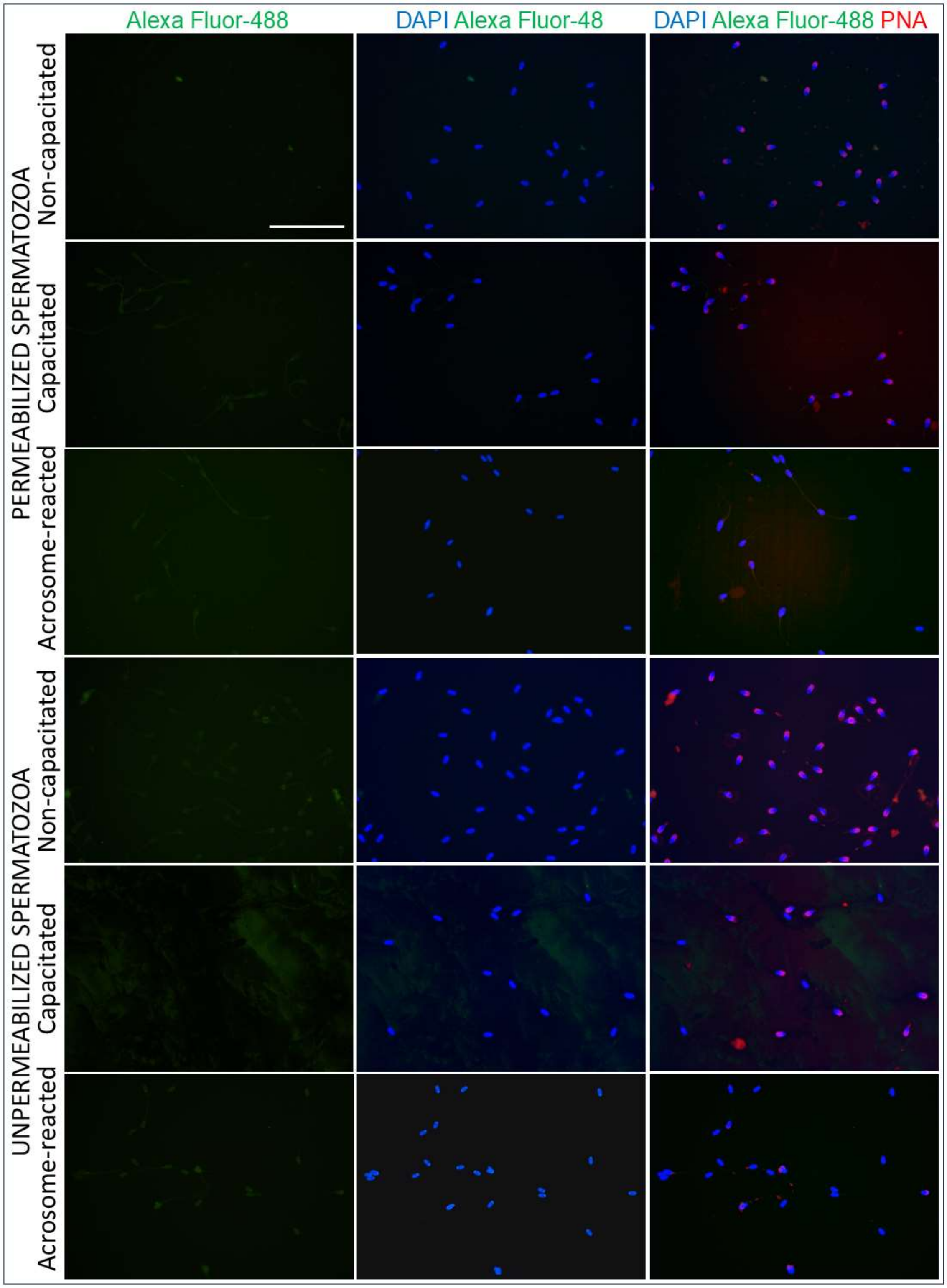

SUPPLEMENTARY FIGURE S3 | Negative controls for Supplementary Figure S2 of permeabilized and unpermeabilized boar spermatozoa at different post-testicular maturation stages. Spermatozoa were incubated with secondary fluorescent goat anti-rabbit antibody Alexa Fluor-488 (green). Acrosomes were stained with lectin PNA (red), and DNA was stained with DAPI (blue). The scale bar represents 50 µm.

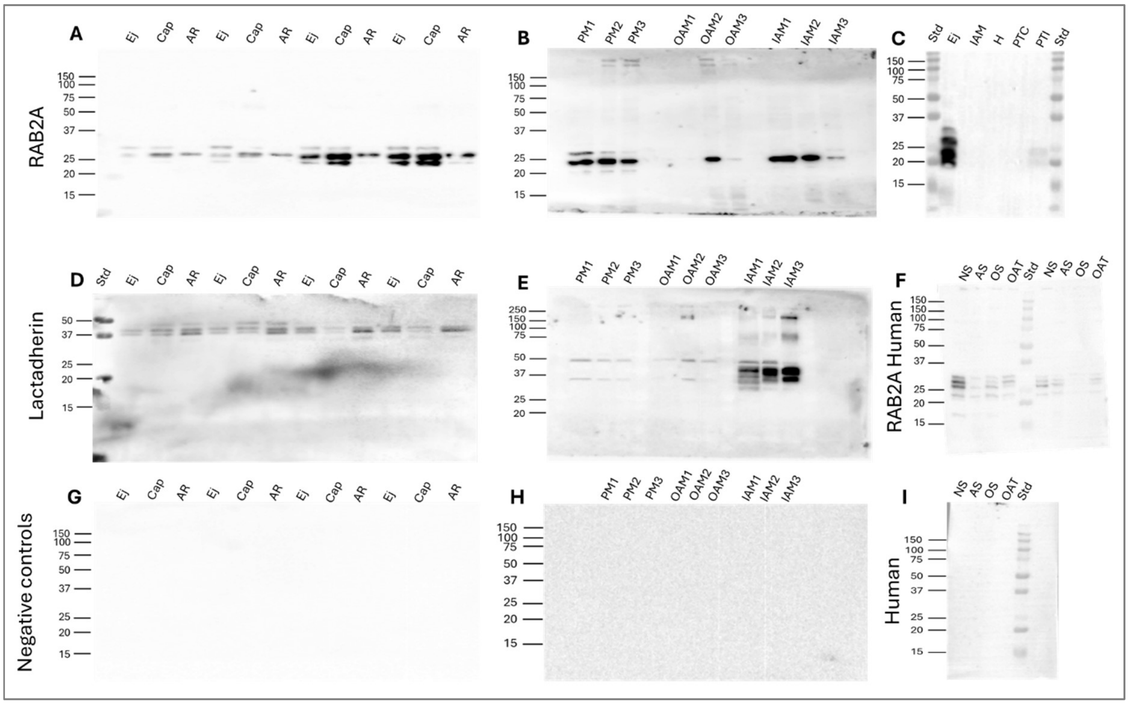

SUPPLEMENTARY FIGURE S4 | Raw data and negative controls for WB analysis. (A) Detection of RAB2A in ejaculated (Ej), capacitated (Cap), and acrosome-reacted (AR) boar spermatozoa (related to Figure 2A); (B, C) Detection of RAB2A in membrane fractions and isolated perinuclear theca (PT) of boar spermatozoa (related to Figure 2C); (D) Detection of lactadherin in Ej, Cap, and AR boar spermatozoa (related to Figure 4A); (E) Detection of lactadherin in membrane fractions of boar spermatozoa (related to Figure 4C); (F) Detection of RAB2A protein in normal and pathological spermatozoa (related to Figure 8C). (G,H,I) Negative controls for Western blot analysis. Membranes were incubated with secondary antibody only, and no nonspecific signal was detected. PM1-3, enriched plasma membrane fractions; OAM1-3, enriched outer acrosomal membrane fractions; IAM1-3, enriched inner acrosomal membrane fractions; H, sperm head fraction; PTC, covalently-bound proteins from PT; PTI, ionically-bound proteins of PT; Std, molecular standards; NS, sperm proteins from normozoospermic ejaculates; AS, sperm proteins from asthenozoospermic ejaculates; OS, sperm proteins from oligozoospermic ejaculates, OAT, sperm proteins from oligoasthenoteratozoospermic ejaculates.

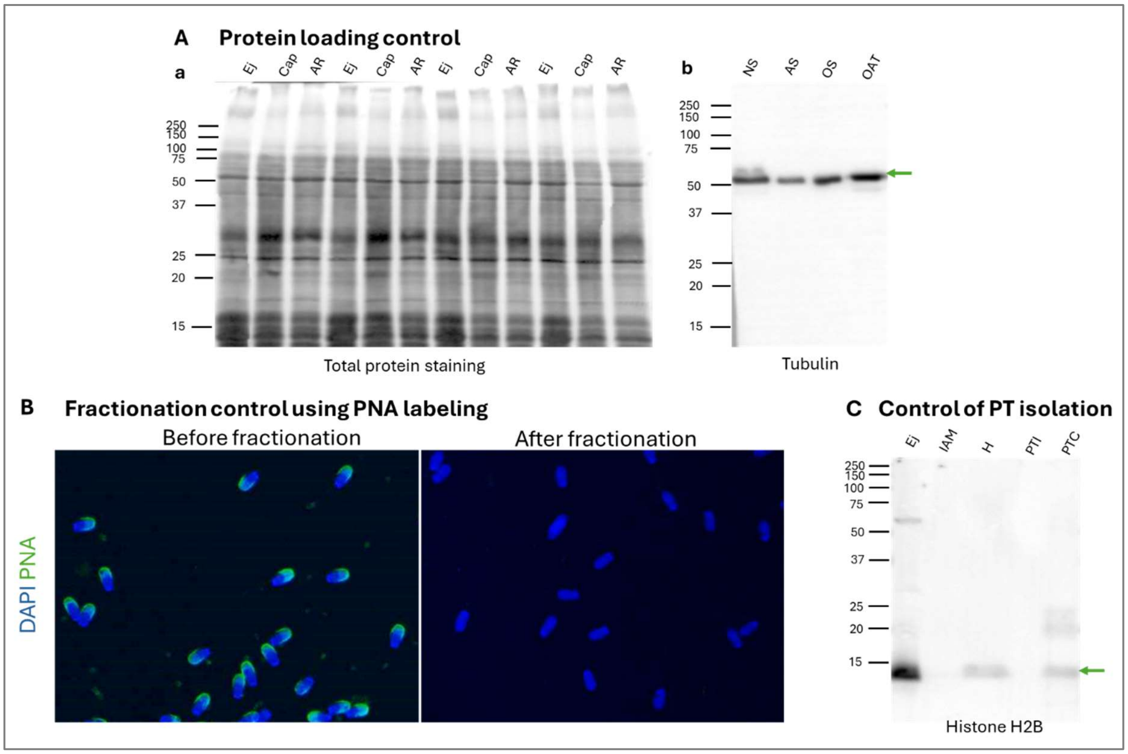

SUPPLEMENTARY FIGURE S5 | Controls for isolations. (A) Loading controls for isolated proteins corresponding to the samples used for densitometric analysis. (a) For boar ejaculated (Ej), capacitated (Cap), and acrosome-reacted (AR) sperm, the membrane was stained with Coomassie Brilliant Blue, showing the protein pattern in each sample. (b) For human sperm, tubulin detection was used as a loading control. (B) Fractionation control was performed using fluorescent lectin labeling of the acrosome with PNA lectin (green). Before fractionation, a signal was detected in the acrosomal region of ejaculated sperm. After fractionation, the signal was lost, and acrosomes were absent in the head fraction containing the attached inner acrosomal membrane. Nuclei are stained with DAPI (blue). (C) Control of isolated perinuclear theca (PT). Histone H2B, which like RAB2A is a component of the PT, was used as a control. Signal was detected in total lysate, in the remaining heads after PT isolation, and in the PT fraction (PTC). A specific signal is indicated by green arrows.

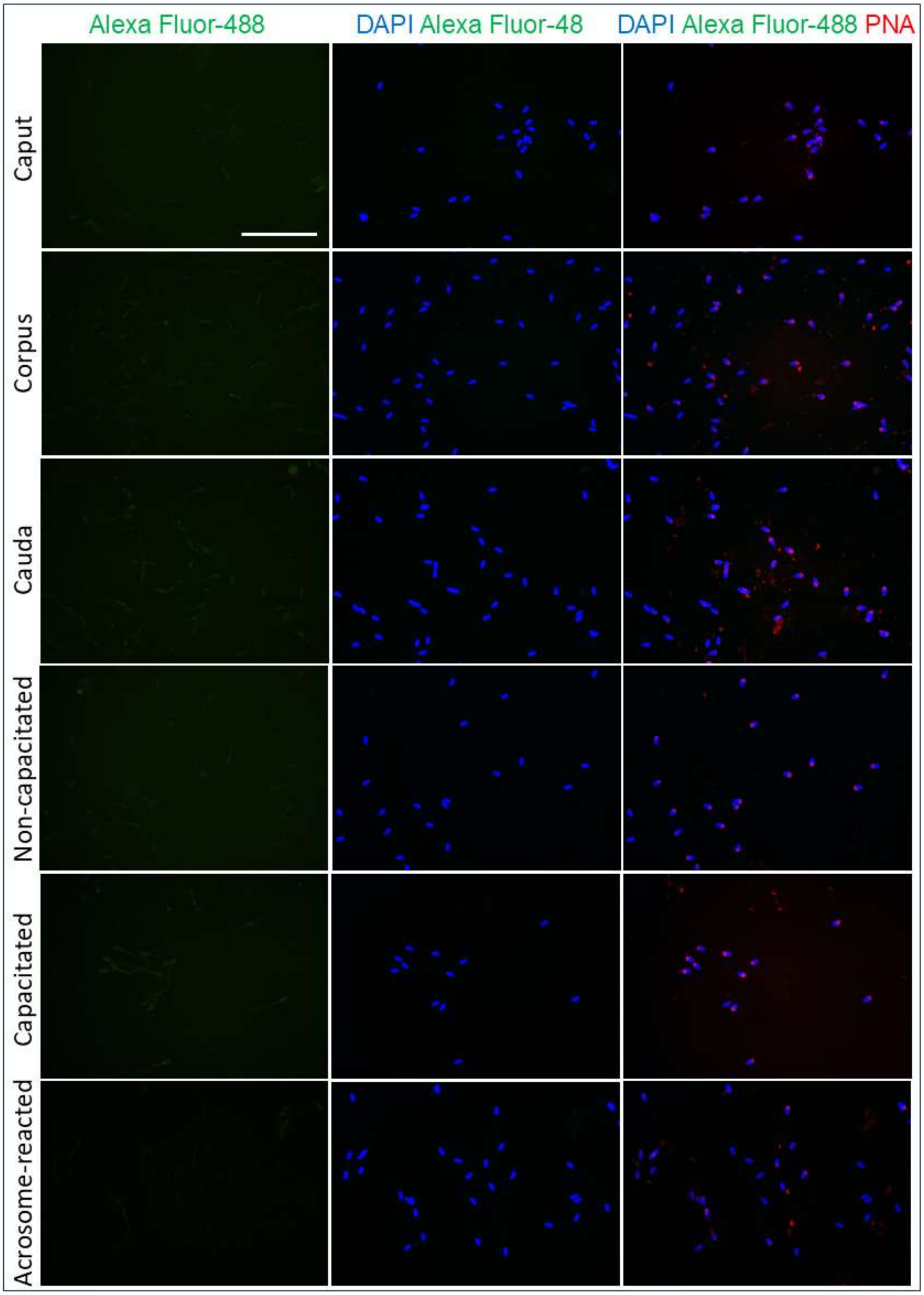

SUPPLEMENTARY FIGURE S6 | Negative controls for Figures 1 and 3 of permeabilized boar spermatozoa at different post-testicular maturation stages. Spermatozoa were incubated with secondary fluorescent goat anti-mouse antibody Alexa Fluor-488 (green). Acrosomes were stained with lectin PNA (red) and DNA was stained with DAPI (blue). The scale bar represents 50 µm.

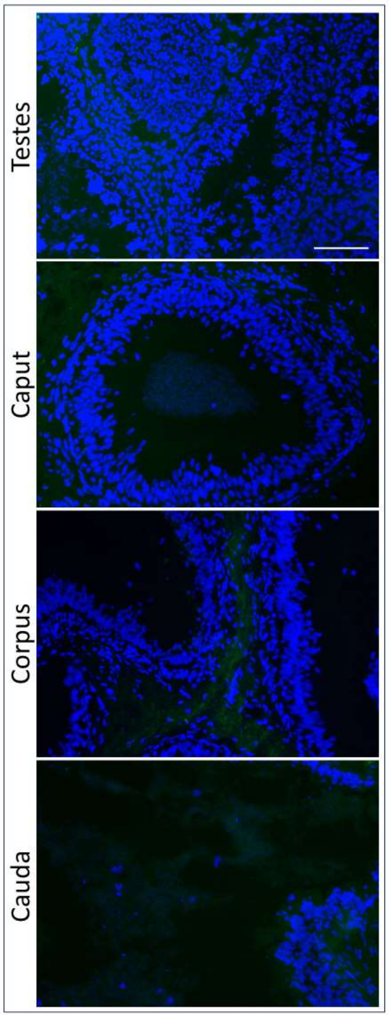

SUPPLEMENTARY FIGURE S7 | Negative controls for Figures 1 and 3 of reproductive tissue sections. Testicular and caput, corpus, and cauda epididymal tissue sections were incubated with secondary fluorescent goat anti-mouse antibody Alexa Fluor-488 (green). DNA was stained with DAPI (blue). The scale bar represents 100 µm.

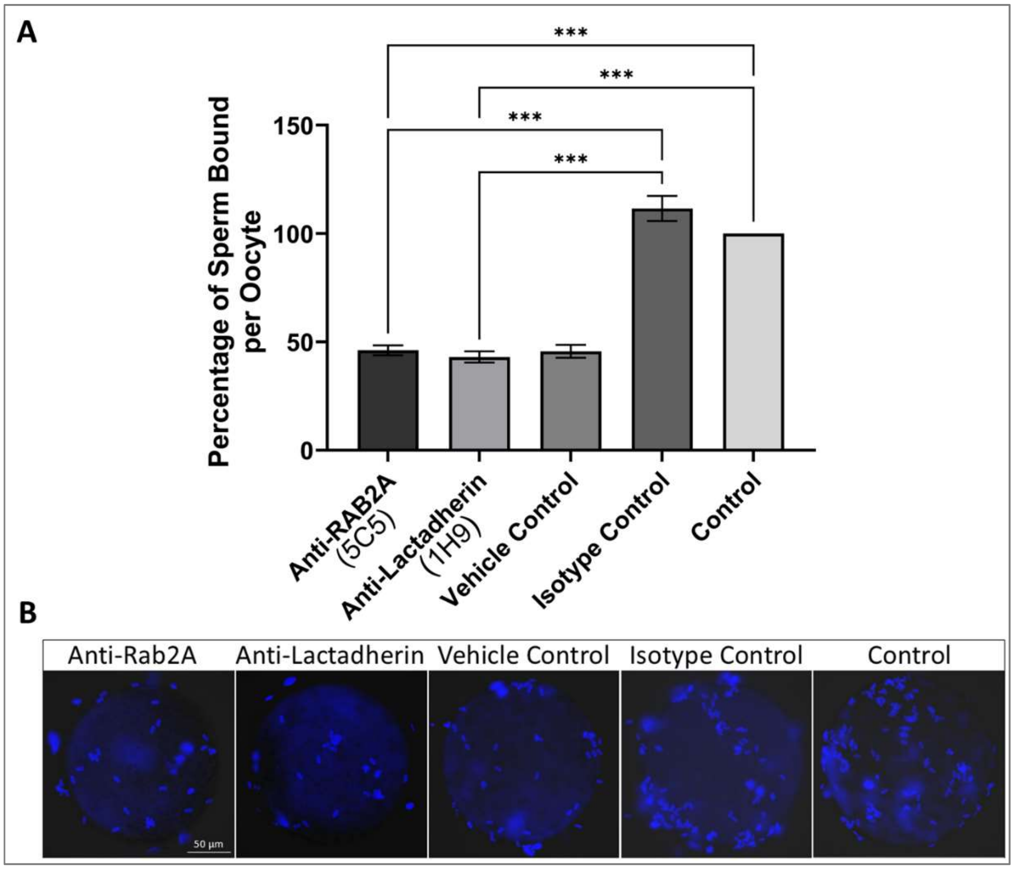

SUPPLEMENTARY FIGURE S8 | Statistical evaluation of the number of sperm bound to the ZP-intact oocytes in *in vitro* binding assays using anti-RAB2A and anti-lactadherin pre-treated spermatozoa. (A) A significant reduction in the number of sperm bound to the ZP-intact oocytes was observed in the anti-RAB2A (N=101) and anti-lactadherin (N=89) groups compared to isotype (N=71) and normal control (N=137), however, no significant reduction was observed compared to vehicle control (N=74), represented by the hybridoma medium, in which the antibodies were prepared and stored, therefore, only experimental groups of anti-RAB2A and anti-lactadherin compared to vehicle control were used in the main text. *** $p < .001$ (B) Representative images of DAPI-stained spermatozoa bound to the ZP-intact oocytes per each group. The scale bar represents 50 µm.

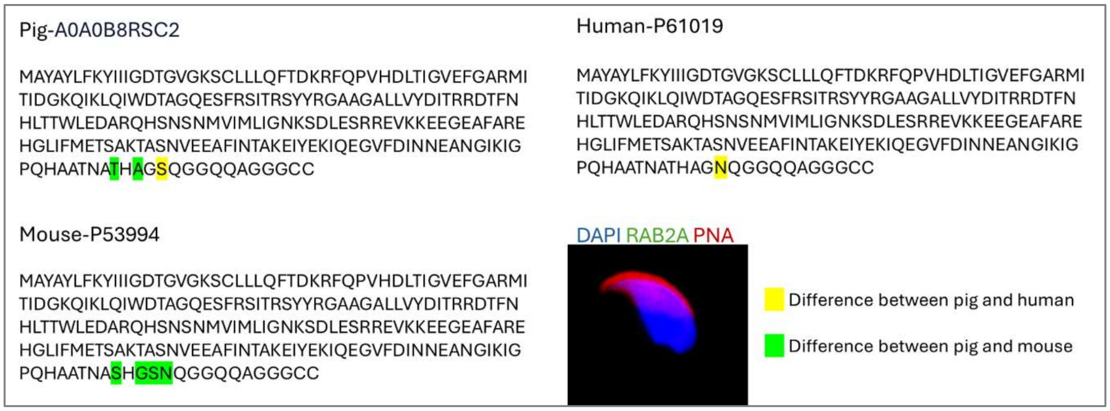

SUPPLEMENTARY FIGURE S9 | Interspecies detection and comparison of the amino acid sequence of the RAB2A protein. The amino acid sequence of porcine RAB2A differs from the human RAB2A protein in only one amino acid (highlighted in yellow), whereas the mouse contains several different amino acids (highlighted in green) near the C-terminal end. Fluorescent labeling for the RAB2A protein with 5C5 antibody on mouse sperm was negative suggesting that the epitope for the 5C5 antibody is located near the C-terminal end.

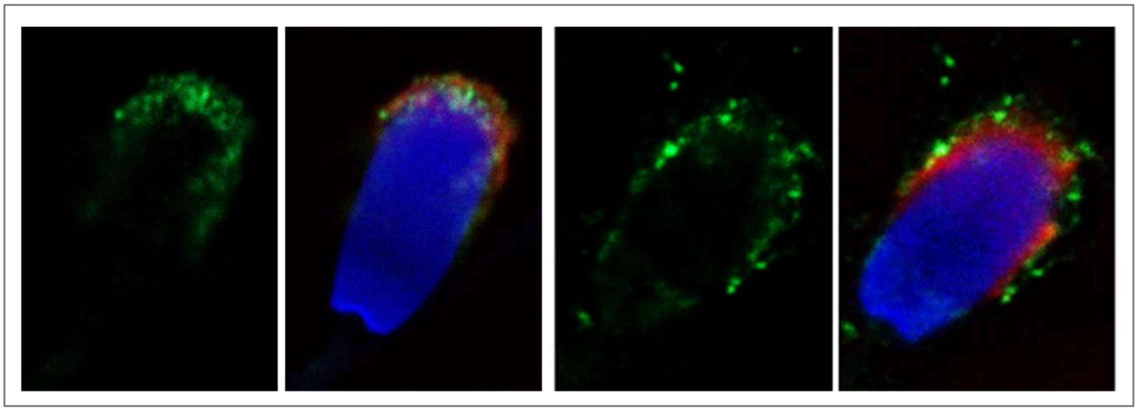

SUPPLEMENTARY FIGURE S10 | Representative RAB2A localization in the acrosome region of unpermeabilized boar spermatozoa showing reorganisation of membranes during ongoing capacitation. At the onset of the acrosomal exocytosis, the RAB2A signal is visible on the acrosome, and as the disruption of the acrosome continues, the signal appears to localize into hybrid vesicles. Spermatozoa were incubated with secondary fluorescent goat anti-mouse antibody Alexa Fluor-488 (green). Acrosomes were stained with lectin PNA (red), and DNA was stained with DAPI (blue).

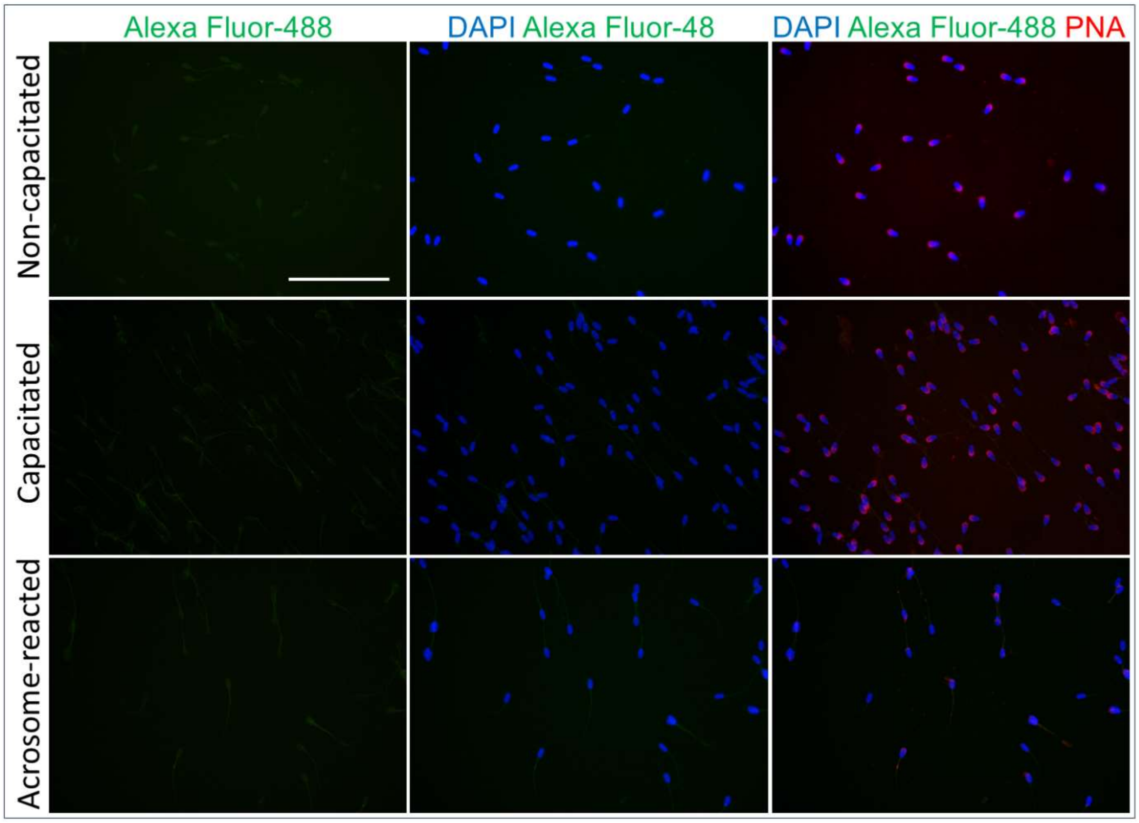

SUPPLEMENTARY FIGURE S11 | Negative controls for Figures 6 and 7 of unpermeabilized boar spermatozoa at different post-testicular maturation stages. Spermatozoa were incubated with secondary fluorescent goat anti-mouse antibody Alexa Fluor-488 (green). Acrosomes were stained with lectin PNA (red), and DNA was stained with DAPI (blue). The scale bar represents 50 µm.

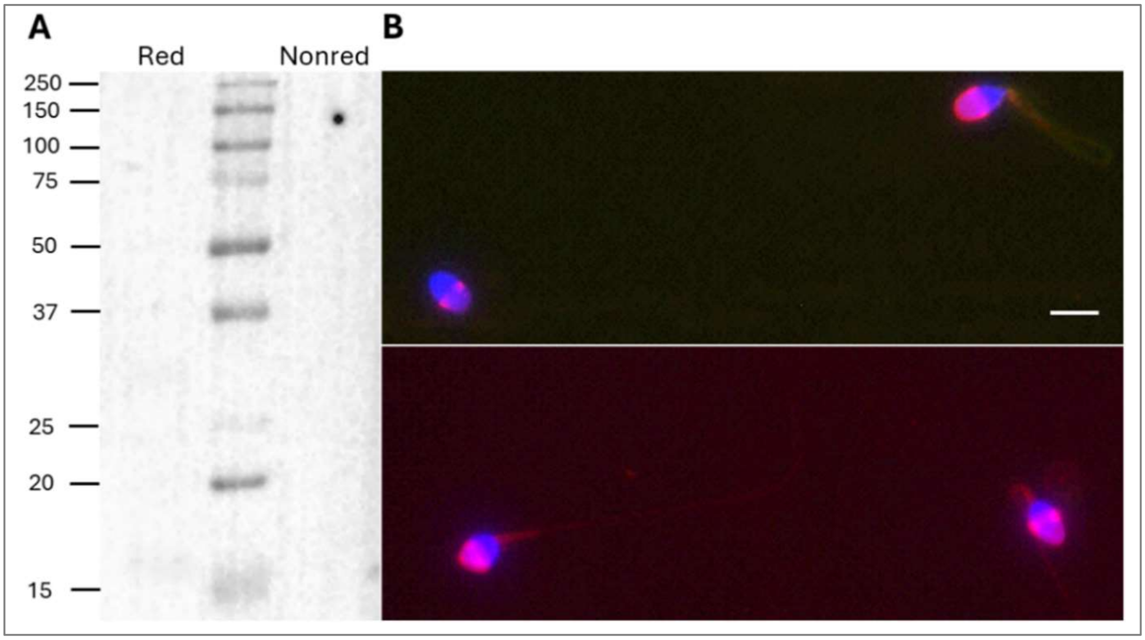

SUPPLEMENTARY FIGURE S12 | Detection and visualization of lactadherin/MFGE8 on human sperm with 1H9 antibody. (A) Negative detection of lactadherin on the human sperm in reduced (Red) and non-reduced (Nonred) conditions by 1H9 antibody. (B) Negative signal after fluorescent labelling of lactadherin with antibody 1H9 on human spermatozoa. The scale bar represents 3 µm.

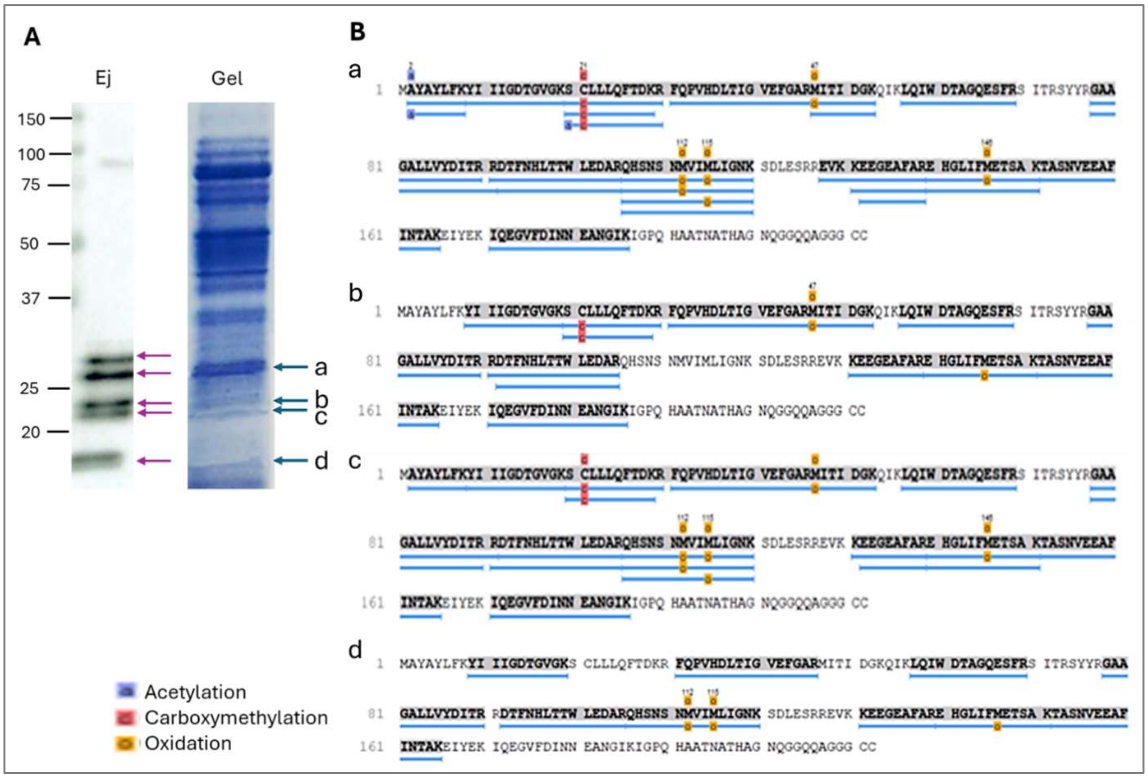

SUPPLEMENTARY FIGURE S13 | Identification of RAB2A in the multibands of human sperm lysate (A) Immunodetection of RAB2A protein (purple arrows) in human ejaculated spermatozoa (Ej) after Western blot and protein profile stained in gel by Coomassie Brilliant Blue, from which the protein bands (a-d) were subjected to mass spectrometry analysis (blue arrows). (B) Mass spectrometry confirmed the presence of RAB2A protein in protein bands labeled with the 5C5 antibody in human sperm lysate. Peptides detected by mass spectrometry are highlighted in gray and underlined with a blue line. Possible post-translational modifications are indicated in colour.
